# Loop-mediated regulation and base flipping drive RNA cleavage by human mitochondrial PNPase

**DOI:** 10.1093/nar/gkaf1296

**Published:** 2025-12-09

**Authors:** Ole Unseld, Hrishikesh Das, B Martin Hällberg

**Affiliations:** Department of Cell and Molecular Biology, Karolinska Institutet, Stockholm 171 77, Sweden; Department of Cell and Molecular Biology, Karolinska Institutet, Stockholm 171 77, Sweden; Department of Cell and Molecular Biology, Karolinska Institutet, Stockholm 171 77, Sweden; CSSB Centre for Structural Systems Biology, c/o DESY, Notkestraße 85, Building 15, Hamburg 22607, Germany

## Abstract

Human polynucleotide phosphorylase (hPNPase), a trimeric exoribonuclease, is crucial for maintaining mitochondrial RNA metabolism, including the regulated degradation of RNA. Mutations in hPNPase have been linked to mitochondrial pathologies, underscoring its importance in mitochondrial RNA homeostasis. Despite this significance, the molecular basis of its catalytic mechanism and the structural consequences of active-site mutations remain poorly understood. We employed high-resolution electron cryo-microscopy to capture three distinct functional states of hPNPase during RNA degradation. In the loading state, flexible loops facilitate the recruitment of the substrate RNA and guide it toward the active site. During the pre-catalytic state, terminal nucleotides reorient within the active site, positioning the RNA backbone for cleavage, which is stabilized by Mg^2+^. Finally, the catalytic state reveals a nucleophilic attack of phosphate on the RNA backbone, mediated by key active-site residues. These results offer a clear biochemical framework for hPNPase-mediated RNA turnover, clarifying its catalytic mechanism and highlighting how active-site integrity is crucial for efficient RNA degradation.

## Graphical Abstract

**Graphical Abstract:**
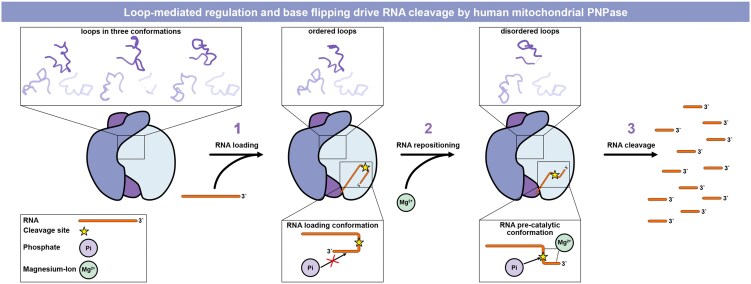

## Introduction

Polynucleotide phosphorylase (PNPase) is a highly conserved 3′–5′ exoribonuclease essential for RNA metabolism in organisms ranging from bacteria to humans [1–4]. In human cells, human polynucleotide phosphorylase (hPNPase) is localized to the mitochondria, where it plays critical roles in maintaining RNA homeostasis through the degradation of mitochondrial RNA antisense transcripts, R-loop processing, and transcription inhibition relief together with the human mitochondrial helicase hSuv3 [5–9]. Mutations in hPNPase were shown to cause various diseases, including hearing loss, neurological disorders, and respiratory chain deficiencies [10–12], underlining the importance of hPNPase for maintaining human mitochondrial functionality.

The architecture for hPNPase is crucial for its RNA degradation capabilities and is highly conserved across multiple species, ranging from human and bacterial PNPases to the archaeal exosome [3, 13–17]. In all forms of life, PNPase catalyzes the phosphorolytic degradation of RNA, utilizing inorganic phosphate as a nucleophile to cleave the RNA phosphodiester bonds and release small RNA fragments with a 5′-diphosphate and a 3′-hydroxyl group [18–20]. Further, the structural arrangement of hPNPase is central to its catalytic function. Three identical subunits assemble into a barrel-shaped trimer with a channel forming a central pore in hPNPase. Each subunit contains two RNase PH-like domains (PH1 and PH2) alongside the K homology (KH) and S1 domains. Together, the six RNase PH domains form a hexameric ring, with the active site located in the PH2 domain [15, 21]. Meanwhile, the KH and S1 domains cap the central pore, creating a scaffold that has been shown to interact with hSuv3 [21]. The central pore of hPNPase, formed by the KH domains and parts of the PH domains, is thought to facilitate RNA threading and, thereby, RNA degradation [15, 21].

Despite significant advances in understanding hPNPase’s domain architecture and physiological importance in humans, critical gaps remain regarding its RNA substrate recognition, catalytic mechanism, and conformational dynamics. Specifically, the role of magnesium ions (Mg²⁺) and phosphate (Pi) in the catalytic mechanism, as well as the structural role of the flexible loops located in the pore in guiding RNA binding and the catalytic mechanism, remains unclear.

Using single-particle cryo-electron microscopy (cryo-EM) and biochemical assays, we examined hPNPase in both its apo and RNA-bound states. Our findings show how RNA and phosphate are coordinated during phosphorolysis, reveal the dynamic rearrangements of its pore loops, and provide a mechanistic basis for RNA turnover and homeostasis in mitochondria. Altogether, these results enhance our understanding of hPNPase’s functional dynamics and underscore its vital role in mitochondrial RNA metabolism.

## Materials and methods

### Cloning and protein expression

The hPNPase gene (amino acids 46–783) was codon optimized for expression in human cell lines and ordered as a gene fragment (IDT) without the N-terminal mitochondrial targeting signal (MTS). The gene was cloned into a pDSG103 vector (IBA) with a C-terminal Twin-Strep-tag (TS) using the HiFi DNA Assembly Cloning Kit (NEB).

### Cell cultivation and transfection

The MEXi-293E cells (IBA) were cultivated and transfected according to the manufacturer’s protocol. In brief, MEXi-293E cells were cultured in MEXi-CM media (IBA) supplemented with 50 mg/mL G-418 (Thermo Fisher Scientific) and 200 mM GlutaMAX (Thermo Fisher Scientific) at 37°C and 5% CO_2_ while shaking at 120 rpm. The cells were subcultured to a cell density of 0.5–0.75 × 10^6^ cells/mL once the cell density reached 1.5–3.0 × 10^6^ cells/ml. For transfection, only cells with a viability above 90% and a density below 3.0 × 10^6^ cells/mL were used. The appropriate amount of cell suspension for 1.5 × 10^6^ cells/mL in a total volume of 15 mL was centrifuged at room temperature and 200 x g for 5 min and resuspended in previously equilibrated MEXi-TM media (IBA) supplemented with 50 mg/mL G-418 (Thermo Fisher Scientific) and 200 mM GlutaMAX (Thermo Fisher Scientific). Next, plasmid DNA was added to a final concentration of 1.5 µg/mL of culture, and the suspension was incubated at 37°C, 5% CO2, and 120 rpm for 10 min. This was followed by the addition of PEI MAX to a concentration of 5 µg/mL of culture and further incubation at 37°C, 5% CO2, and 120 rpm for 3 h. Finally, 15 mL MEXi-CM media (IBA) supplemented with 50 mg/mL G-418 (Thermo Fisher Scientific) and 200 mM GlutaMAX (Thermo Fisher Scientific) was added to a final volume of 30 ml. Once the transfected cells reached a density of 3 × 10^6^ cells/mL, 95 ml of MEXi-CM media (IBA) supplemented with 50 mg/ml G-418 (Thermo Fisher Scientific) and 200 mM GlutaMAX (Thermo Fisher Scientific) was added. The cells were harvested 7 days after transfection by centrifugation at 200 × *g*, and stored at −80°C.

### Protein purification

For biochemical and structural analysis, hPNPase with a C-terminal Twin-Strep-tag (TS) was recombinantly expressed and purified. First, MEXi-293E cells (IBA) from a 150 mL suspension culture were thawed on ice and resuspended in lysis buffer (20 mM HEPES adjusted to pH 8.0 with 10 M NaOH, 300 mM NaCl, 1 mM MgCl_2_, 1 × Pierce protease inhibitor ethylenediaminetetraacetic acid (EDTA)-free (Thermo Fisher Scientific), 1 mM phenylmethylsulfonyl fluoride (PMSF), 1 mM dithiothreitol (DTT), and 10% glycerol). Cell lysis was performed by sonication (15 s pulse, 15 s rest, 20% amplitude, 10 cycles). The lysate was clarified by centrifugation at 13 000 × *g* for 20 min at 4°C.

Strep-Tactin Superflow XT 4Flow beads (IBA) were equilibrated with five column volumes (CV) of lysis buffer before application of the clarified lysate. The beads were washed with 15 CV of lysis buffer. Protein was eluted using 2 CV of elution buffer (20 mM HEPES adjusted to pH 8.0 with 10 M NaOH, 300 mM NaCl, 1 mM MgCl_2_, 1 × Pierce protease inhibitor EDTA-free (Thermo Fisher Scientific), 1 mM PMSF, 1 mM DTT, 10% glycerol, and 50 mM biotin). The eluted protein was concentrated to a final volume of 0.9 mL and loaded onto a Superdex 200 10/30 GL size exclusion chromatography column (SEC; Cytiva) pre-equilibrated with SEC buffer (20 mM HEPES adjusted to pH 8.0 with 10 M NaOH, 150 mM NaCl, 1 mM MgCl_2_, 1 mM DTT, and 1% glycerol). The peak fractions containing purified protein were analyzed using 4%–12% sodium dodecyl sulfate–polyacrylamide gel electrophoresis (SDS–PAGE) (Supplementary Fig. S1; Invitrogen), pooled and further concentrated, aliquoted, and stored at −80°C for downstream applications.

### RNA degradation assays

The *in vitro* RNA degradation assays were performed to biochemically characterize RNA degradation by hPNPase using different substrates and reaction conditions. The reactions were performed in RNA degradation buffer (10 mM HEPES adjusted to pH 8.0 with 10 M NaOH, 50 mM NaCl, 2 mM MgCl_2_, 2 mM DTT, and 2 mM NaH_2_PO_4_) if not specified differently in the figure legend. The reaction mixtures contained 0.2 µM hPNPase-TS and 1 µM of the respective RNA substrates, as indicated in the gel images (Supplementary Table S1), and were incubated at 37°C. At defined time points, 2 µl aliquots were withdrawn, and the reactions were terminated by adding an equal volume of 2 × TBU sample loading buffer (89 mM Tris–HCl, pH 8.3, 89 mM boric acid, 2 mM EDTA, 4 M urea, and 12% Ficoll PM 400), followed by the addition of 1 µl proteinase K (RNase/DNase free, Thermo Fisher Scientific, 20 mg/mL). Samples were incubated at 50°C for 30 min to degrade proteins. The processed samples were resolved on a 15% TBE-Urea PAGE (BioRad Criterion or Invitrogen), run at a constant 150 V for 45 min.

### RNA-DNA competition assay

To *in vitro* characterize whether DNA can compete with RNA for binding, an RNA-DNA competition assay was performed. For competition, either 1:1 or 1:5 molar ratios of RNA:DNA were used (Supplementary Table S1). The RNA concentrations were kept constant at 1 μM, and the DNA concentrations varied between 1 and 5 μM. The reaction, termination, and TBE-Urea PAGE conditions were as described for the RNA degradation assays.

### RNA mass spectrometry

The reaction mixtures for RNA mass spectrometry (RNA-MS) analysis were prepared in RNA degradation buffer (10 mM HEPES adjusted to pH 8.0 with 10 M NaOH, 50 mM NaCl, 2 mM DTT, 2 mM MgCl_2_, and 0.2 mM NaH_2_PO_4_). Reactions contained 1 µM hPNPase-TS and 5 µM RNA (Supplementary Table S1), maintaining a 1:5 protein:RNA molar ratio, in a total reaction volume of 20 µl. Incubations were carried out at 37°C. Reactions were terminated by heating the samples to 90°C for 1 min, followed by snap freezing in liquid nitrogen and storage at −80°C. RNA-MS experiments were performed in negative electrospray ionization mode using an ESI-QTOF instrument (Waters). Chromatographic separation was achieved on an ACQUITY BEH C18 column with mobile phases consisting of water and acetonitrile (ACN), each supplemented with 5 mM ammonium acetate (pH 4.5). An 8-min gradient was employed for separation. The MS setup accommodated multiply charged species up to at least 50 kDa, and all analyses included an internal standard for quality control.

For each RNA-MS sample, 0.5 µl aliquots were withdrawn from the heat-terminated reactions and analyzed using the previously described TBE-Urea PAGE in the RNA degradation section.

### Cryo-EM grid preparation

The general procedure for *in vitro* reconstitution is described here, with specific variations outlined for individual samples in subsequent sections. For reconstitution, RNA degradation buffer (10 mM HEPES adjusted to pH 8.0 with 10 M NaOH, 50 mM NaCl, 2 mM MgCl_2_, 2 mM DTT, and 2 mM NaH_2_PO_4_) was mixed with 1.5 µM hPNPase-TS and a 5 × molar excess of RNA substrate, and the reaction mixture was incubated at 37°C. Before cryo-EM grid preparation, grids were glow-discharged with 35 mA for 1 min on the back and 35 mA for 2 min on the front using an EMS 100X (Electron Microscopy Sciences) glow-discharge unit. Grids used were UltrAuFoil Gold 200 mesh (R 1.2/1.3 geometry; Quantifoil Micro Tools GmbH). 3.5 μL aliquots of sample solutions were applied to the grids, and the grids with the sample were then vitrified in a Vitrobot Mk IV (Thermo Fisher Scientific) at 4°C and 100% humidity [blot 5 s, blot force 3, 595 filter paper (Ted Pella Inc.)].

#### hPNPase apo

hPNPase was incubated in RNA-degradation buffer on ice, and 3.5 μL aliquots of sample solutions were applied to the grids.

#### hPNPase bound to PT5 RNA

RNA substrate containing five (PT5) phosphorothioate bonds (denoted with an asterisk in the sequences below) was used for *in vitro* reconstitution of the RNA-loading state. The reaction mixture was incubated at 37°C for 20 min.

PNP_RNA_PT5_short sequence:

5′-rArCrArCrArArCrArCrArArCrArCrArCrArCrCrArCrArCrArCrA*rC*rA*rU*rA*rArArArCrArArArArCrA -3′

#### hPNPase bound to RNA

The protein buffer was modified to contain 10 mM Na_2_SO_4_ and 0.1 mM NaH_2_PO_4,_ replacing the standard 2 mM NaH_2_PO_4_. The sample was reconstituted by incubation at 37°C for 30 min. The reaction mixture included an RNA substrate with the name and sequence:

FAM_PNP_RNA_long:

/56-FAM/rCrArCrArArCrArCrArCrArCrArCrCrArCrArCrArCrArCrArUrArArArArCrArArArArCrArGrCrUrArCrGrCrCrArUrCrCrUrCrCrCrCrCrCrArArUrCrUrA.

### Cryo-EM data collection

Cryo-EM data were collected using EPU (Thermo Fisher Scientific) on a Krios G3i transmission electron microscope (Thermo Fisher Scientific) operated at 300 kV in the Karolinska Institutet 3D-EM facility. Micrographs were acquired in nanoprobe 165kX EF-TEM SA mode at a pixel size of 0.5076 Å using a K3 BioQuantum direct electron detector with a slit width of 10 eV. Each exposure was 1.8–2 s, divided into 56–59 fractions, with a total fluence of 46–58 e⁻/Å².

### Initial single-particle data processing

Motion correction, dose weighting, contrast transfer function (CTF) estimation, and Fourier cropping (to 1.0152 Å/px) for a total of 6182 for apo, 20 309 for RNA-loading state, and 4465 for pre-catalytic state movies were performed in real-time using CryoSPARC Live v4.6.2 [22]. Micrographs with an estimated resolution better than 5 Å and an under-focus range of 0.2–4 µm were kept for further processing. For all datasets, the dose-weighted and motion-corrected averages were processed in CryoSPARC v4.6.2 [22]. Initial particle picking was performed using the blob picker with a size threshold of 90–130 Å, followed by 2D classification. High-quality 2D classes were used for *ab initio* reconstruction and subsequent heterogeneous refinement. Particles from well-resolved heterogeneous classes were re-extracted and used for training a Topaz model [23]. The trained model was used to improve particle picking, followed by 2D classification, *ab initio* reconstruction, and multiple rounds of heterogeneous refinement to exclude suboptimal particles.

### hPNPase Apo single-particle data processing

Good particles from heterogeneous refinement were used for non-uniform refinement (C1). Next, global and local CTF refinements were performed, followed by reference-based motion correction (RBMC). Subsequently, focused 3D classifications with a mask spanning the central pore of hPNPase were performed to identify particles with similar loop conformation. The final reconstruction employed local refinements (C1) with a mask that covered the trimer. For further details, see Supplementary Fig. S2.

### hPNPase RNA loading state single-particle data processing

After non-uniform refinement (C1) using good particles from the heterogeneous refinement, global and local CTF refinements and RBMC were performed. Next, a mask spanning all three active sites was utilized in combination with 3D variability analysis and volume alignment tools to align the RNA-containing particles to the same active site. To prevent the alignment of the RNA to other protomers, only local refinements (C1) with a mask spanning the whole hPNPase trimer were performed. Subsequently, the particles were exported to RELION-5 beta [24–26] using pyem [27] and 3D auto-refinement (C1), and global and local CTF refinements were performed. Multiple rounds of 3D classification focused on the RNA-containing protomer without image alignment, and varying T values between 40 and 100 were used to remove particles without RNA. The final particle set was imported into cryoSPARC v4.6.2 [22], and global and local CTF refinements were performed. The final reconstruction used local refinements (C1) with a mask covering the trimer. For further details, see Supplementary Fig. S3. To further improve the density of the RNA extending toward the bottom of hPNPase, the particles were re-extracted with a pixel size of 1.59 Å, and 3D variability analysis, focused on the RNA, was performed in cryoSPARC v4.7.1 [22]. Particles with extending RNA density were selected and extracted with 1.0152 Å pixel size used for local refinement (C1) utilizing a mask covering the trimer. For further details, see Supplementary Fig. S4.

### hPNPase pre-catalytic state single-particle data processing

High-quality particles from heterogeneous refinement were used for non-uniform refinement, followed by global and local CTF refinements and RBMC. To align RNA-containing particles in the same active site, a mask spanning all three active sites in combination with 3D classification, 3D variability analysis, and volume alignment tools was used. To prevent the realignment of the RNA-containing particles to other protomers, local refinement (C1) with a mask spanning the whole trimer was used hereafter. Next, the particles were exported to RELION-5 beta [24–26] using pyem [27], and 3D auto-refinement (C1) and global and local CTF refinements were performed. Multiple rounds of 3D classification with a mask covering the RNA-containing protomer and *T*-values ranging from 30 to 80 were performed to select RNA-containing particles. The final particle stack was imported into cryoSPARC v4.6.2 [22], within which global and local CTF refinements were performed, and the orientation distribution was rebalanced. The final reconstruction employed local refinements (C1) with a mask that covered the trimer. For further details, see Supplementary Fig. S5. The RNA density of the reconstruction after RNA alignment (rotated particle stack), resembled a heterogeneous state between the RNA-bound loading and pre-catalytic state. To isolate individual states, we used guided 3D classification without image alignments in RELION-5 [24–26]. Particles with RNA density in the loading state were imported into cryoSPARC v4.7.1 [22] and refined using local refinement (C1) with a mask covering the trimer and a reference volume lacking density for the active site to reduce bias during classification. Next, the particles were exported with pyem [27], and non-guided 3D classification without image alignments was performed in RELION-5 [24–26]. The best particles were imported into cryoSPARC v4.7.1 [22], and local refinement (C1) with a mask covering the trimer was used as the final reconstruction method. For further details, see Supplementary Fig. S6.

### Postprocessing, model building, and validation

For molecular model building, an AlphaFold2 [28] model (AF-Q8TCS8-F1-v4) was used as a starting model. The models were built, refined, and validated using Isolde [29], Coot [30], PHENIX [31], and MolProbity [32]. Furthermore, cryoSPARC v4.6.2 or v4.7.1 [22], 3DFSC [33], and BlocRes [34] were used for cryo-EM reconstruction validation. UCSF ChimeraX [35] was used for visualization, generation of structural figures, and mol maps. EMReady v2.1 [36] was used to postprocess the reconstruction to improve visualization.

### Sequence alignments

Sequence alignments between hPNPase and PNPases of other species were performed with PROMALS3D [37] and ESPript 3.0 [38].

## Results

### Trimeric human PNPase bound to Phosphate (Pi) in the active site

**Figure 1.**
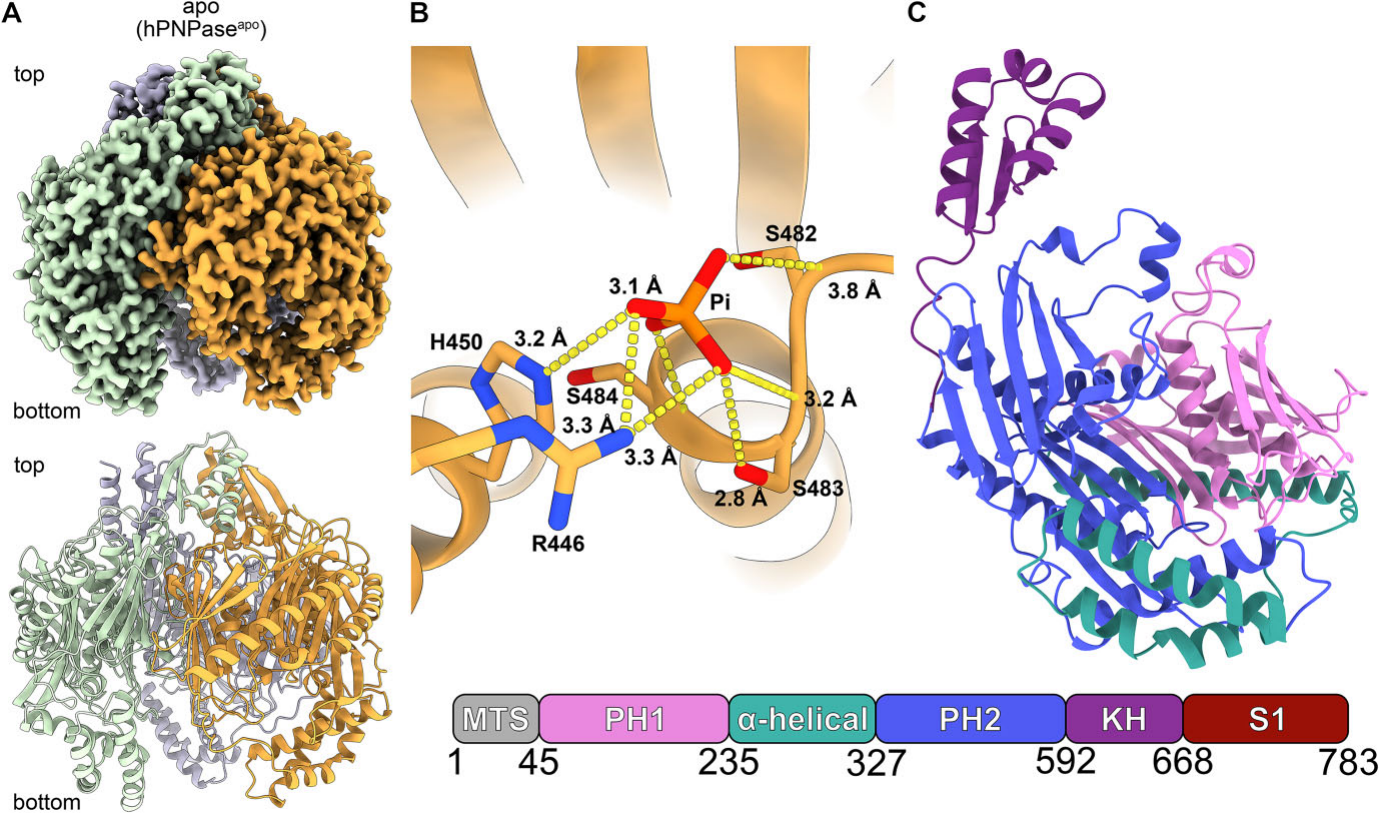
Structural overview of human PNPase in the apo state. (**A**) Cryo-EM map and cartoon representation of trimeric hPNPase in the apo state (hPNPase^apo^; PDB code: 9NJC). Each protomer is colored individually in orange, green, and blue. (**B**) The phosphate (Pi) binding site in hPNPase^apo^ PH2 domain active site. Interactions are indicated in dashed lines. (**C**) Domain architecture of hPNPase alongside a cartoon representation of hPNPase protomer. The catalytic core, comprising PH1, PH2, and α-helical domains, is depicted in pink, blue, and green, respectively. The KH domain is in violet. The unmodeled MTS and S1 domain are colored in gray and red, respectively.

To understand the structural basis of hPNPase-mediated RNA degradation in human mitochondria, we determined the structure of hPNPase bound to a phosphate ion in its active site to an overall resolution of 2.4 Å using single-particle cryo-EM (Fig. 1A and B, Supplementary Fig. S2, and Supplementary Table S2). The structure revealed a homotrimeric assembly forming a barrel-like structure formed by the KH, PH1/2, and α-helical domains (Fig. 1A–C). The phosphate ion is present in each protomer and coordinated by S482-484, H450, and R446, located in the PH2 domain (Fig. 1B and Supplementary Fig. S7A–C). The KH-domains form a pore at the top of the trimeric assembly (hereafter called pore). Our co-substrate-bound structure (with Pi) is similar to a previous apo crystal structure of hPNPase (1.4 Å^2^ RMSD; [15]).

### Human PNPase is a 3′–5′ exoribonuclease using phosphate (Pi) as a co-substrate

To establish the conditions required to solve the structure of hPNPase bound to an RNA substrate, we performed biochemical characterization using various RNA substrates, including phosphorothioate (PT)-modified RNA, a modification commonly used to inhibit nuclease cleavage [39, 40]. Our and previously published work shows that hPNPase activity strictly required both Mg²⁺ and phosphate ions (Supplementary Fig. S8A) [15, 20], and phosphate was consumed in the exonucleolytic reaction, consistent with phosphate being a co-substrate rather than a cofactor (Supplementary Fig. S8B). Furthermore, sulfate cannot replace phosphate as a co-substrate and halts RNA degradation (Supplementary Fig. S8C). To further challenge hPNPase RNA degradation capabilities, we next used substrates with five PT bonds or DNA nucleotides. hPNPase was able to cleave those substrates, although with a markedly reduced cleavage rate (Supplementary Fig. S8D). We next validated whether the reduced cleavage activity was a result of nonphysiological RNA binding of the PT RNA by using a substrate with only a single non-PT modified ribonucleotide. hPNPase degraded the substrate with almost similar efficiency as the positive control (Supplementary Fig. S8E), indicating a limited effect of PT-modified RNA on hPNPase’s ability to bind and degrade PT-modified RNA substrates.

### Trimeric human PNPase bound to RNA in the active site

**Figure 2.**
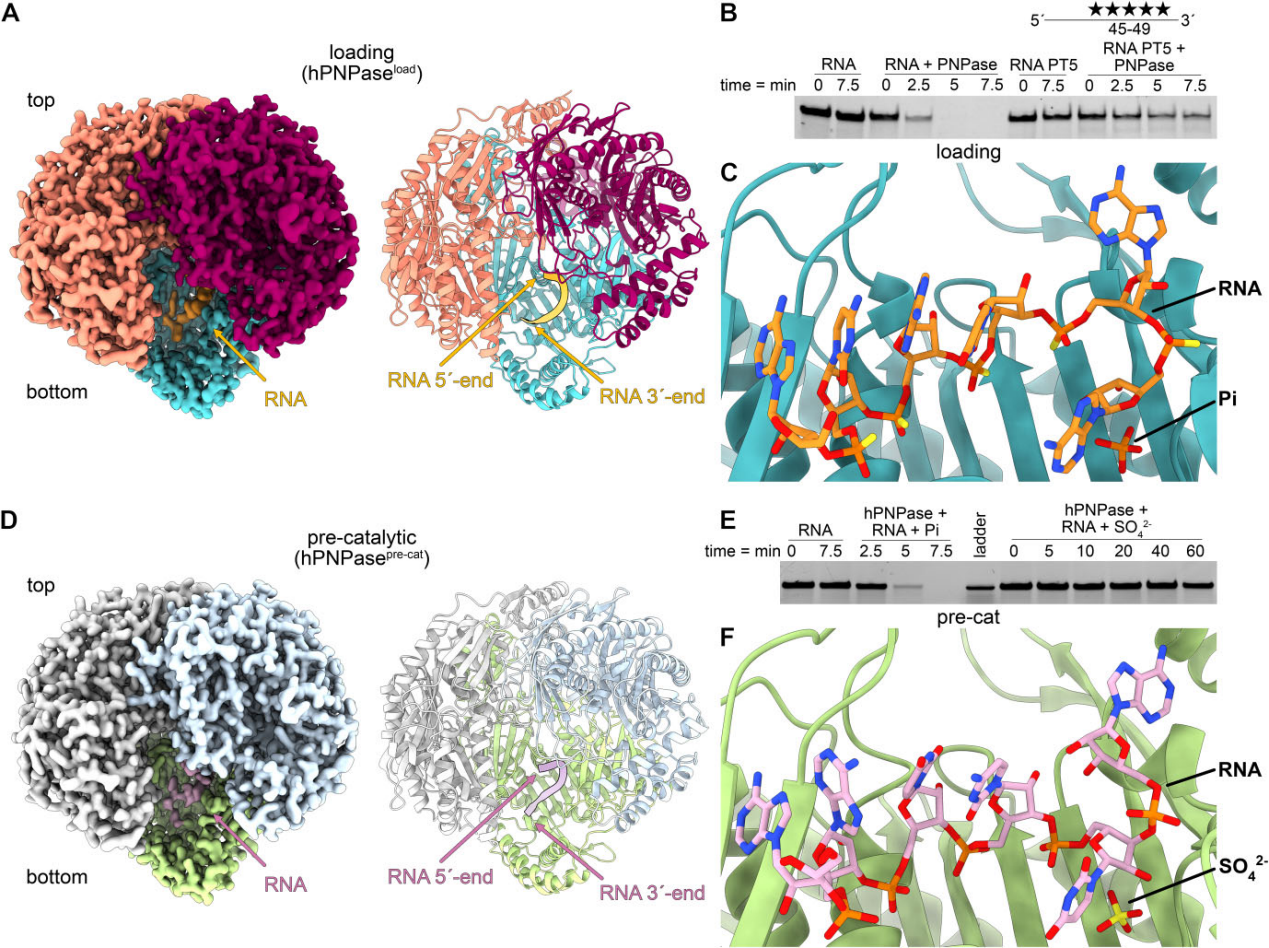
Structural overview of human PNPase in RNA-bound states. (**A**) Cryo-EM map and cartoon representation of trimeric hPNPase in RNA loading state (hPNPase^load^; PDB code: 9NO0). Each protomer and active site-bound RNA are colored individually in red, violet, blue, and orange. The 3′- and 5′-ends of the RNA are labeled in the cartoon representation. (**B**) hPNPase RNA degradation assay using five times phosphorothioate-modified (PT5) RNA. The RNA degradation was assessed by 15% TBE-Urea PAGE (full TBE-Urea-PAGE in Supplementary Fig. S8D). (**C**) RNA substrate and the phosphate (Pi) co-substrate bound in the hPNPase^load^ active site. (**D**) Cryo-EM map and cartoon representation of trimeric hPNPase in RNA pre-catalytic state (hPNPase^pre-cat^; PDB code: 9NJB). Each protomer and active site-bound RNA is colored individually in gray, blue, green, and violet. The 3′- and 5′-ends of the RNA are labeled in the cartoon representation. (**E**) hPNPase RNA degradation assay using 2 mM SO_4_^2−^ and RNA. The RNA degradation was assessed by 15% TBE-Urea PAGE (full TBE-Urea-PAGE in Supplementary Fig. S8C). (**F**) RNA substrate and the co-substrate mimic sulfate (SO_4_^2−^) bound in the hPNPase^pre-cat^ active site.

To elucidate the RNA substrate binding and degradation mechanism by hPNPase, we determined hPNPase bound to RNA under two different conditions in which the turnover rate is reduced or abolished. In the condition that gave rise to what we will henceforth call the loading state structure (hPNPase^load^; 2.1 Å; Fig. 2A–C; Supplementary Fig. S3 and  Supplementary Table S2), we used an RNA substrate in which five phosphodiester bonds had been replaced with PT bonds (PT5), which reduces but does not abolish hPNPase cleavage activity (Fig. 2B). To capture a state closer to the catalytic state, we replaced phosphate with sulfate and obtained what we will henceforth call the pre-catalytic state structure (hPNPase^pre-cat^; 2.2 Å; Fig. 2D–F; Supplementary Fig. S5 and  Supplementary Table S2). Both the hPNPase^load^ and hPNPase^pre-cat^ structures reveal a six-nucleotide (nt) long RNA in the active site of hPNPase (Fig. 2C and F). Furthermore, the active-site phosphate and sulfate in the hPNPase^load^ and hPNPase^pre-cat^ structures, respectively, retain their relative positions compared to hPNPase^apo^ (Figs 1B and 2C and F). We further validated the hPNPase^load^ reconstruction (hPNPase^load-A^; PDB:9NO0) through an additional 3D classification of the pre-catalytic state particles (Supplementary Fig. S6). This additional hPNPase^load^ structure (hPNPase^load-C^; PDB: 9XYI), derived from a subset of the hPNPase^pre-cat^ particle set (PDB code: 9NJB), shows a similar RNA binding (Supplementary Fig. S9A and B), PH1 and PH2 domain loop (Supplementary Fig. S9C–F), and pore architecture as the initial hPNPase^load^ structure (hPNPase^load-A^; PDB: 9NO0) (Supplementary Fig. S9G–I) with an RMSD of 0.21 Å^2^.

### Structural basis for RNA substrate binding in human trimeric PNPase

**Figure 3.**
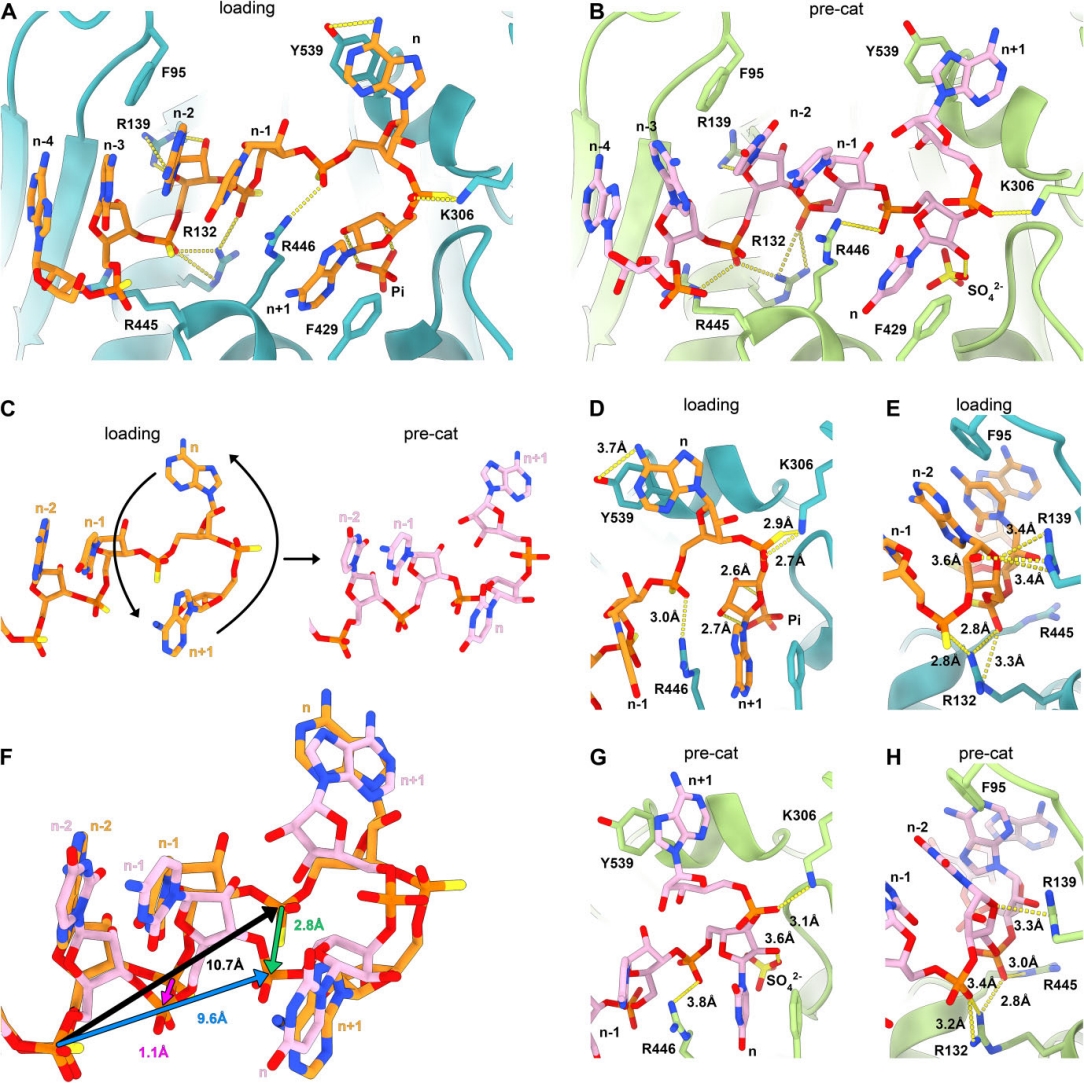
RNA binding to hPNPase active site in loading and pre-catalytic state. RNA bound to hPNPase active site in loading (hPNPase^load^; PDB code: 9NO0) (**A**) or (**B**) pre-catalytic (hPNPase^pre-cat^; PDB code: 9NJB) conformation. Protein-RNA interactions are indicated as dashed lines. (**C**) The 3′-terminal bases in positions *n* and *n* + 1 relative to the scissile phosphate are flipped by 180° along the horizontal axis, allowing for transition from the RNA loading to the pre-catalytic conformation. (**D, E**) hPNPase^load^ key protein-RNA interactions, indicated in dashed lines. (**F**) Superposition of RNA in the binding state and pre-catalytic state. The 3′-terminal base flip shifts the RNA phosphate-ribose backbone downwards and backwards relative to the loading state.(**G, H**) hPNPase^pre-cat^ key protein-RNA interactions, indicated with dashed lines.

RNA binding near and within the hPNPase active site is mediated by sequence-independent interactions, facilitating the degradation of a broad range of mitochondrial RNA substrates. The protein-RNA interactions involve the key residues R132, R446, and K306, which form salt bridges with the RNA phosphate backbone, and F95, F429, Y539, R139, and R446, which contribute to base stacking interactions (Fig. 3A and B). In both RNA binding states, hPNPase forms only a few interactions unique to RNA, such as a hydrogen bond between R139 and the 2′-OH in position n-2. Hence, the enzyme’s overall substrate specificity for RNA over DNA arises from reaction chemistry (e.g. 2′-OH dependency).

Biochemical assays are consistent with this model. DNA, although not a substrate, competes for the binding site and reduces RNA turnover (Supplementary Fig. S10A and B), and 2′-*O*-methylation markedly lowers the cleavage rate, highlighting the requirement for a free 2′-hydroxyl group, only present in RNA (Supplementary Fig. S10C).

In the hPNPase^load^ structure, the RNA adopts a U-shaped conformation (Fig. 3C), which maximizes interactions with the hPNPase active site and places the 2′- and 3′-hydroxyl groups near the phosphate co-substrate (Fig. 3D and E). In contrast, in the hPNPase^pre-cat^ structure, the two 3′-terminal nucleotides *n* and *n* + 1 are flipped, giving the RNA an S-shaped conformation (Fig. 3C). This rearrangement in hPNPase^pre-cat^ shifts the scissile phosphate closer to the active site (Fig. 3F) and prevents the 3′-hydroxyl from contacting the phosphate (Fig. 3G). As a result, interactions between the RNA and R446/K306 (Fig. 3G) as well as with R132/R139 (Fig. 3H) in the active site are weakened, leading to a slightly less stable RNA binding, which will improve later product release.

### Structural basis of human PNPase RNA cleavage

**Figure 4.**
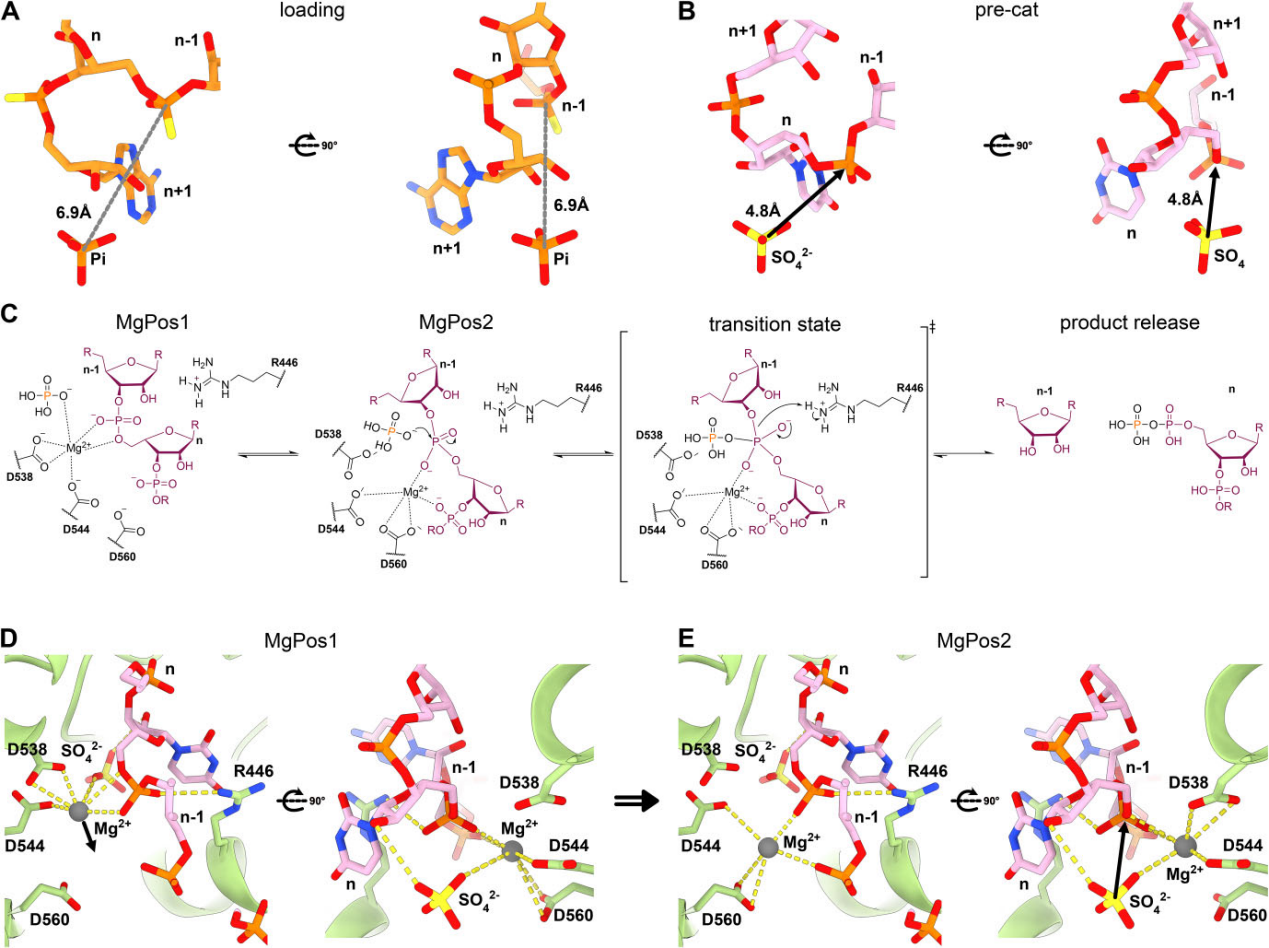
The RNA phosphorolysis cleavage mechanism.(**A**) RNA loading conformation (hPNPase^load^; PDB code: 9NO0): 3′-terminal ribose is positioned between the phosphate (Pi) co-substrate and the scissile phosphate, thereby preventing an effective nucleophilic attack. (**B**) RNA pre-catalytic conformation (hPNPase^pre-cat^; PDB code: 9NJB): a 3′-terminal base flip positions the scissile phosphate in proximity to the phosphate co-substrate mimicking sulfate (SO_4_^2−^), and this would enable a nucleophilic attack.(**C**) Reaction mechanism scheme for RNA cleavage by hPNPase. The RNA substrate is colored violet, and the nucleophilic attacking phosphate (Pi) co-substrate is colored orange. The magnesium ion (Mg^2+^) has dual functions: positioning the phosphate (SO_4_^2−^ here) for nucleophilic attack on the scissile phosphate in position 1 (**D**) and stabilizing the cleavage transition state in position 2 (**E**). Interactions are indicated with dashed lines.

Combining our structural and biochemical work points to a two-step exonucleolytic reaction mechanism for hPNPase that allows the degradation of various RNAs. In this two-step process, RNA is initially bound in the RNA-loading state (hPNPase^load^), where the scissile phosphate (n) is positioned too far from the phosphate co-substrate in the active site for nucleophilic attack and is further sterically hindered by the terminal ribose (Fig. 4A). The two terminal nucleotides flip in the second step, transitioning into the precatalytic state, hPNPase^pre-cat^ (Fig. 3C). Now, the scissile phosphate is positioned near the co-substrate phosphate without steric hindrance (Fig. 4B), and the RNA is locked into position through base stacking of R446/F429 against base n and a salt bridge and base stacking through K306 and Y539 with base n + 1 (Fig. 3B and G). The hPNPase^pre-cat^ state is additionally stabilized by the interaction of R446 with the scissile phosphate and an Mg²⁺-ion coordinated through D538, D544, D560, and the RNA backbone phosphate in position n and n-1 (Fig. 4C and D). In our reconstructions of hPNPase^pre-cat^, we find the magnesium in two approximately equally occupied positions (MgPos1 and MgPos2, Supplementary Fig. S11A–C). In the MgPos1, the magnesium ion – in conjunction with the 2′-hydroxyl group of the ribose – aligns and stabilizes the co-substrate phosphate for nucleophilic attack on the scissile phosphate (Fig. 4C and D; Supplementary Fig. S11D). Upon nucleophilic attack of the phosphate on the RNA scissile bond, the magnesium ion is sterically forced by the phosphate into the less favored and transient MgPos2, closer to D560 and RNA backbone phosphate in position n and n-1 (Fig. 4C and 4E; Supplementary Fig. S11E–G). Together with R446, the MgPos2 magnesium stabilizes the transition state (Fig. 4C and E), and reaction products are likely released after protonation of the transition state by R446 or water (Fig. 4C).

Next, we probed the substrate tolerance and degradation mechanism of hPNPase using defined RNA substrates. Single non-cleavable PT modifications at positions 1, 20, or 21 (counted from the 3′ end) did not measurably affect the cleavage rate (Supplementary Fig. S12A and B). In contrast, substrates bearing five or ten PT modifications, or a five DNA nucleotides stretch (RNA-DNA-hybrid), were degraded with reduced efficiency (Supplementary Figs S8D and 12C). An RNA containing three 8-oxoguanosine (8-oxo-rG) residues, reported to stall *Escherichia coli* PNPase [41], was still processed by hPNPase with only a modest reduction in rate (Supplementary Fig. S12D). RNA-MS of the cleavage products indicated predominant dinucleotide release from the 3′-end (Supplementary Fig. S13A–D) and identified a trinucleotide as the minimal 5′-product (Supplementary Fig. S14A–C) in accordance with the observation that single-base modifications did not alter hPNPase cleavage rate.

Collectively, these findings illustrate how hPNPase coordinates RNA loading, base flipping, and cleavage to achieve efficient, 3′–5′ phosphorolytic RNA degradation into small RNA fragments.

### Non-structured RNA is guided through the bottom into the active site

**Figure 5.**
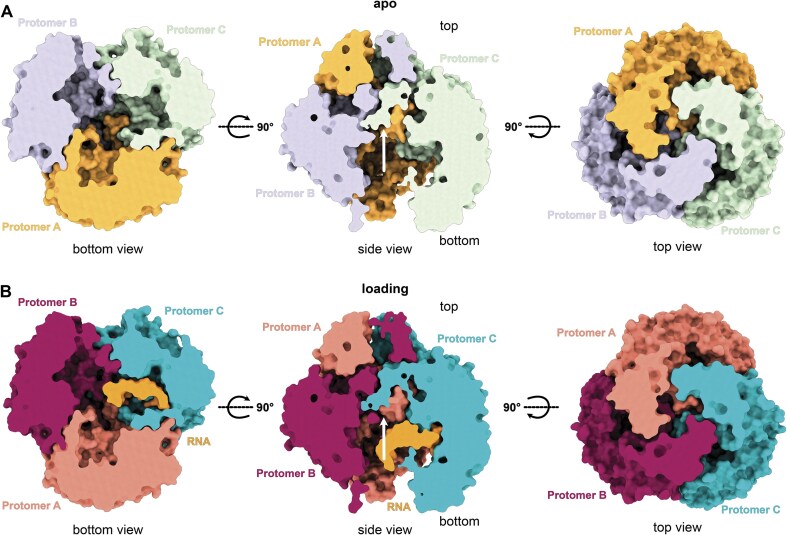
Loops block the hPNPase trimer top central pore, and RNA access to the active site is only available through the hPNPase trimer bottom.(**A**) Surface representation of trimeric hPNPase^apo^ structure in loop conformation 1 (hPNPase^apo-loop-conf1^; PDB code: 9NJC) and (**B**) the RNA-bound loading state (hPNPase^load^; PDB code: 9XZF). Views are from the bottom, the side, and the top, respectively. Each protomer is colored individually in violet, green, and orange for hPNPase^apo-loop-conf1,^ and each protomer and RNA are colored in violet, blue, red, and orange for hPNPase^load^. White arrows point toward the pore occluding PH1 and PH2 domain loops in the side views.

It was previously proposed that, similar to bacterial PNPase, RNA is threaded through the central pore of trimeric hPNPase into the active site [15]. In contrast, our cryo-EM reconstruction of hPNPase^apo^ shows that the pore is occluded by loops extending from the PH1 (residues 104–124) and PH2 (residues 405–423) domains, which are oriented toward the center of the pore and restrict the available space (Fig. 5A). In the hPNPase^load^ reconstructions, these loops remain in a similar conformation to hPNPase^apo^, preventing RNA from entering through the pore (Fig. 5B and Supplementary Fig. S15A–F; RMSD 2.2 Å^2^). Further classification of hPNPase^load^ (hPNPase^load-A^; PDB code: 9NO0) allowed us to resolve additional nucleotides of the highly flexible RNA 5′-end, resulting in an additional hPNPase^load^ reconstruction (hPNPase^load-B^; PDB code: 9XZF; Supplementary Fig. S4). Those hPNPase^load^ structures, alongside hPNPase^pre-cat^ (PDB code: 9NJB), show RNA density extending toward the bottom of hPNPase (Supplementary Fig. S16A) and an occluded pore in all electron density maps (Supplementary Fig. S16B). This indicates that at least non-structured and linear single-stranded RNA accesses the catalytic site from the bottom of the trimeric assembly instead of through the pore in the top of the trimeric assembly.

### Central pore loops allosterically regulate the hPNPase active sites

**Figure 6.**
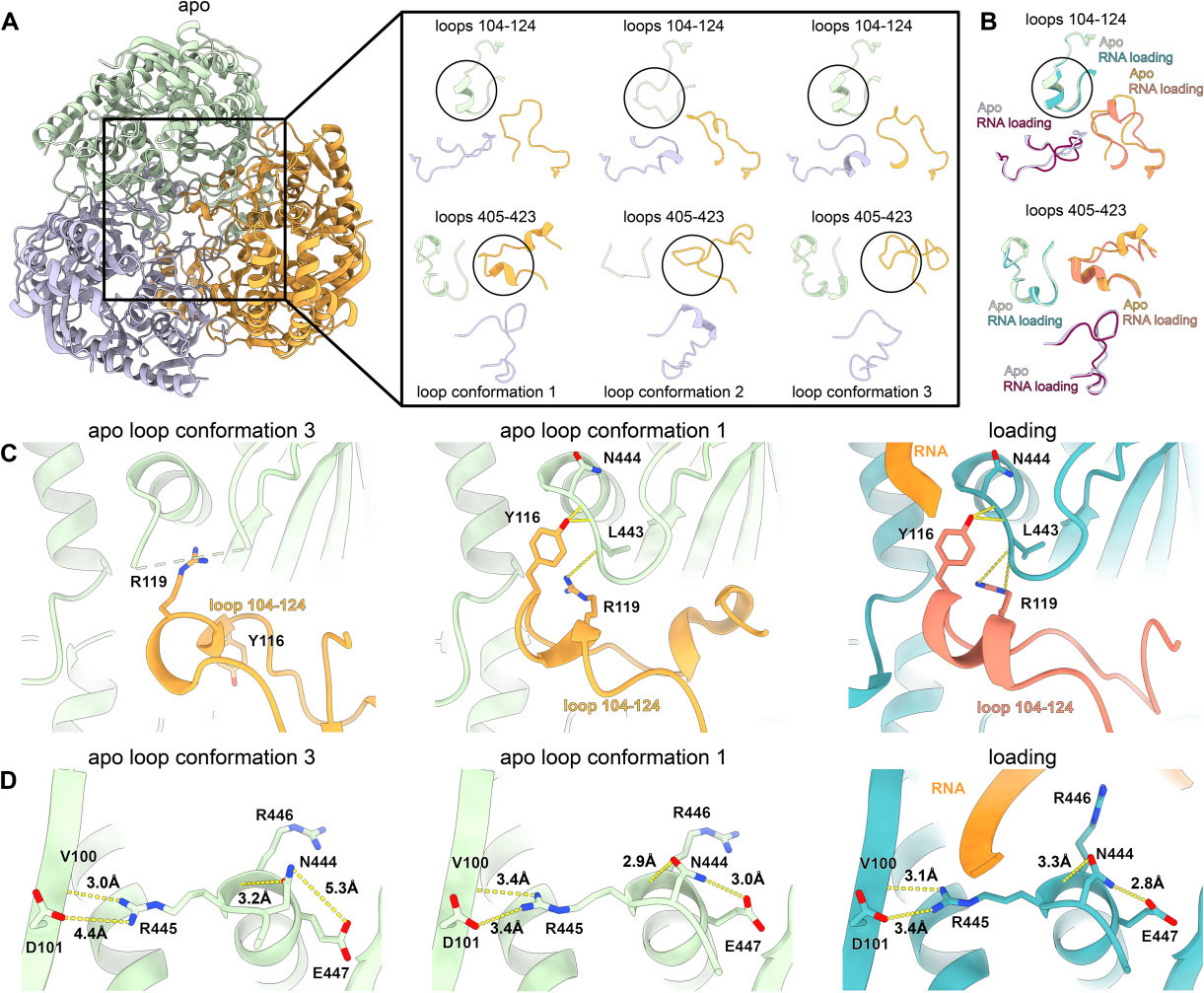
Loops in the central pore allosterically regulate hPNPase active site. (**A**) Close-up view of the pore loops spanning PH1 (residue 104–124) and PH2 (residue 405–423) domains of each protomer for trimeric hPNPase. The apo loop conformations 1 (hPNPase^apo-loop-conf1^; 41.1% of final particles; PDB code: 9NJC), 2 (hPNPase^apo-loop-conf2^; 26.4% of final particles; PDB code: 9NJE), and 3 (hPNPase^apo-loop-conf3^; 32.4% of final particles; PDB code: 9NJD) found in the hPNPase^apo^ structure are shown. Each protomer is colored individually in green, blue, and orange. (**B**) Comparison between loop conformation in RNA loading state (hPNPase^load^; PDB code 9NJO) and hPNPase^apo-loop-conf1^. (**C**) Interaction between loops 104–124 with the adjacent active site for hPNPase^apo-loop-conf3^, hPNPase^apo-loop-conf1^, and hPNPase^load^. Interactions are indicated in dashed lines. (**D**) Close-up on hPNPase^apo-loop-conf3^, hPNPase^apo-loop-conf1^, and hPNPase^load^ active site. Interactions are indicated with dashed lines.

Our cryo-EM reconstructions of hPNPase reveal that the loop architecture and organization depend on the RNA binding state. For the hPNPase^apo^, we observe three distinct asymmetric assemblies of PH1 (residues 104–124) and PH2 (residues 405–423) domain loop conformations throughout 3D classification (Fig. 6A and Supplementary Fig. S2), henceforth called hPNPase^apo-loop-conf1^, hPNPase^apo-loop-conf2^, and hPNPase^apo-loop-conf3^. In contrast to hPNPase^apo^, our analysis shows conformational changes in the loops upon RNA binding in hPNPase^load^ and hPNPase^pre-cat^ (Fig. 6B and Supplementary Fig. S17A).

Differences between hPNPase^apo-loop-conf1^, hPNPase^apo-loop-conf3,^ and hPNPase^load^ reveal changes in interactions between the PH1 loop residues 104–124 with the active site. Only hPNPase^apo-loop-conf1^and the hPNPase^load^ allow a neighboring protomer’s PH1 loop to adopt an α-helical structure extending toward the active site (Fig. 6C). This enables interactions between Y116/R119 (chain B) with L443/N444 (chain C), facilitating R445 to form a stable salt bridge with D101 (chain C), thereby stabilizing the active site (Fig. 6D). These interactions are coupled with the side-chain repositioning of N444, which strengthens its hydrogen-bonding with E447 and R446 (Fig. 6D). Importantly, this stabilization may be necessary as hPNPase^apo^ orients R446 toward the phosphate binding site (Fig. 1B and Supplementary Fig. S17B), a conformation incompatible with RNA binding due to steric clashes (Supplementary Fig. S17B). Active site stabilization and N444 repositioning may allow for the upward reorientation of R446, facilitating efficient RNA entry. Steric constraints limit this rearrangement; only one active site can be stabilized by a neighboring protomer’s PH1 domain loop to prevent steric clashes (Supplementary Fig. S17C). This explains why hPNPase utilizes a single protomer per catalytic cycle. This is consistent with our observation that RNA density is only present in one protomer of the hPNPase^load^ cryo-EM map (Supplementary Fig. S17D). Transition to hPNPase^pre-cat^ initiates a more disordered conformation in all loops (Supplementary Fig. S17A), likely promoting faster product release and priming the enzyme for subsequent catalytic cycles.

## Discussion

Our structural analysis reveals how human mitochondrial PNPase (hPNPase) catalyzes RNA degradation through a phosphorolytic mechanism with 3′–5′ directionality. By capturing three distinct functional states—hPNPase^apo^, hPNPase^load^, and hPNPase^pre-cat^—we demonstrate that non-structured RNA can enter the enzyme through the basal opening rather than the central pore. Our findings elucidate both the allosteric regulation mediated by flexible pore loops and the precise catalytic mechanism underlying RNA degradation. The structural and biochemical data support a two-step mechanism: first, RNA adopts a loading conformation that favors 3′–5′ degradation (hPNPase^load^), followed by a 180° flip of the two 3′-terminal bases that positions the scissile phosphate for nucleophilic attack (hPNPase^pre-cat^).

The sequence-independent RNA recognition mechanism enables hPNPase to process diverse mitochondrial RNA substrates, including R-loops, antisense transcripts, and 7S RNA [7–9]. To achieve 3′-end specificity, hPNPase initially binds the RNA’s 3′-end in the loading conformation, hPNPase^load^ (Fig. 3A). While this arrangement maximizes enzyme-RNA interactions (Fig. 3D and E), it strategically positions the scissile bond away from the phosphate co-substrate, preventing premature cleavage (Fig. 4A). The subsequent flipping of the two 3′-terminal nucleotides, into hPNPase^pre-cat^ (Fig. 3B and C), creates the precise geometry required for in-line phosphorolysis (Fig. 4B), with catalysis dependent on both an essential Mg²⁺ ion and R446, previously identified as critical for activity (Fig. 4C–E; [20]).

Interestingly, the hPNPase^load^ state can accommodate only one nucleotide beyond the scissile bond due to steric constraints (Fig. 2C). Nevertheless, we show that hPNPase can process substrates containing multiple non-cleavable nucleotides (Supplementary Figs. S8D, S10C, and S12C). Therefore, we propose that, despite its 3′-end preference, hPNPase can alternatively bind and process RNA directly in the pre-catalytic conformation (which lacks these steric limitations; Fig. 2F), though with reduced binding affinity and catalytic efficiency. This flexibility parallels the ability of bacterial PNPase to navigate sequence-internal obstacles when processing RNA-DNA hybrid substrates [42].

While phosphorolytic cleavage mechanisms have been proposed for archaeal exosomes and bacterial PNPases, with RNA binding mediated through highly conserved residues (Supplementary Fig. S18) [3, 16, 42], hPNPase exhibits a distinct active-site geometry. Specifically, in comparison to bacterial PNPases, hPNPase contains an amino acid insertion near the active site (Supplementary Fig. S18 and 19A–C) and displays a shifted β-sheet in the PH2 domain (Supplementary Fig. S19D–F). Additionally, the α-helical domain is repositioned toward the RNA substrate (Supplementary Fig. S19G–I). These structural adaptations result in a bent RNA conformation (Supplementary Fig. S20A) rather than the linear arrangement seen in other systems (Supplementary Fig. S20B). This bent conformation is stabilized through additional interactions with K306 of the α-helical domain and Y539, a residue conserved only in eukaryotes (Supplementary Fig. S18 and 20C–E).

These structural distinctions explain several unique properties of hPNPase compared to *E. coli* PNPase: its ability to efficiently degrade 8-oxo-rG modified RNA, its production of oligonucleotide rather than mononucleotide products, and its reduced polyadenylation activity (Supplementary Fig. S12D and 13A and B) [20, 41, 43]. These adaptations likely evolved in response to the specialized role of PNPase in eukaryotic mitochondria and the oxidative stress environment [44].

The Mg²⁺ ion in hPNPase^pre-cat^ serves dual catalytic roles by occupying two transient positions (Fig. 4D and E; Supplementary Fig. S11D–G). In MgPos1, it aligns the attacking phosphate with the 2′-OH group (Fig. 4C and D; Supplementary Fig. S11D). Upon nucleophilic attack of the phosphate, the Mg^2+^ ion is forced into the less favorable MgPos2, where it works with R446 to stabilize the transition state (Fig. 4C and E; Supplementary Fig. S11E).

In the structure presented here, Pi was substituted with sulfate to abolish cleavage while preserving co-substrate binding. Under these nonreactive conditions, the catalytic Mg^2+^ ion transitions between both MgPos1 and the less-favored but transition-state-stabilizing MgPos2, appearing as a single elongated density in our cryo-EM reconstruction (Supplementary Fig. S11A–C). The absence of Mg²⁺ in the loading state and its appearance following nucleotide flipping suggest that Mg²⁺ binding is either coupled to or actively drives the conformational change required for catalysis. Consistent with previous mutational analyses, the evolutionarily conserved residues D544, D538, and D560 are essential for proper Mg²⁺ coordination (Fig. 4D and E and Supplementary Fig. S11D and E) [20, 45]. Moreover, the requirement for the 2′-OH group to orient the phosphate co-substrate explains hPNPase’s selectivity for RNA over DNA substrates (Fig. 4D and E; Supplementary Fig. S10A).

The PH loops constitute an important regulatory mechanism. Our cryo-EM analysis revealed three distinct PH-loop conformations in hPNPase^apo^ (designated hPNPase^apo-loop-conf1-3^). Only hPNPase ^apo-loop-conf1^, which structurally resembles hPNPase^load^ (Supplementary Fig. S15A–F), permits a neighboring PH1 loop to adopt the α-helical structure necessary for active-site stabilization (Fig. 6C). This conformation enables Y116 and R119 to interact with L443 and N444, allowing R445—previously identified as functionally important—to form a stabilizing salt bridge with D101. Notably, since R445 does not directly contact the RNA substrate, the reduced activity observed in R445 mutants [20, 45] likely results from indirect active-site destabilization rather than impaired RNA binding. Steric constraints restrict this stabilizing interaction to a single protomer (Supplementary Fig. S17C), explaining the asymmetric RNA density we observe (Supplementary Fig. S17D) and supporting a model of sequential catalytic turnover with only one active site engaged per cycle. During the transition to hPNPase^pre-cat^, the loops adopt a more disordered conformation (Supplementary Fig. S17A), likely facilitating product release and preparing the enzyme for subsequent catalytic cycles. This dynamic PH-loop behavior, absent in bacterial PNPases, may provide a regulatory interface for mitochondrial interaction partners such as the helicase hSuv3 [4, 8, 21, 46].

A striking difference between hPNPase and bacterial PNPase, despite their sequence similarity (Supplementary Fig. S18), lies in their pore architecture and RNA entry routes. Our structures demonstrate that non-structured RNA can enter hPNPase through the basal opening (Fig. 5B and Supplementary Fig. 16A), contrasting with *E. coli* PNPase (ecPNPase), where RNA accesses the active site via the central pore [14, 41]. This difference arises from distinct KH-domain and PH-domain loop organizations. In both hPNPase^apo^ and hPNPase^load^, the PH1 loops extend to pore center rather than toward the KH domain as in ecPNPase (Supplementary Figs S9C–I, 15A–F, and 21A–F), creating a steric barrier to pore entry already in hPNPase^apo^. Furthermore, hPNPase adopts a more compact KH domain conformation compared to its bacterial counterpart, further constricting the pore (Supplementary Fig. S21G and H). These structural features collectively restrict hPNPase’s central channel, directing single-stranded RNA through the bottom opening.

In conclusion, our work provides a comprehensive molecular framework for understanding hPNPase-mediated RNA degradation in human mitochondria, revealing how structural adaptations have evolved to meet the specific demands of mitochondrial RNA metabolism.

## Supplementary Material

gkaf1296_Supplemental_File

## Data Availability

The coordinates have been deposited in the Protein Data Bank (PDB) with accession codes PDB-ID: 9NO0 (hPNPase^load-A^, also referred to as hPNPase^load^), 9XZF (hPNPase^load-B^, also referred to as hPNPase^load^), 9XYI (hPNPase^load-C^, also referred to as hPNPase^load^), 9NJB (hPNPase^pre-cat^), 9NJC (hPNPase^apo-loop-conf1^), 9NJE (hPNPase^apo-loop-conf2^), and 9NJD (hPNPase^apo-loop-conf3^). The cryo-EM maps have been deposited in the Electron Microscopy Data Bank (EMDB) with accession codes EMD-49590 (hPNPase^load-A^, also referred to as hPNPase^load^), EMD-72352 (hPNPase^load-B^, also referred to as hPNPase^load^), EMD-72335 (hPNPase^load-C^, also referred to as hPNPase^load^), EMD-49590 (hPNPase^load^), EMD-49478 (hPNPase^pre-cat^), EMD-49479 (hPNPase^apo-loop-conf1^), EMD-49481 (hPNPase^apo-loop-conf2^), and EMD-49480 (hPNPase^apo-loop-conf3^). The raw cryo-EM movies, final particle stacks, and final particle location, giving rise to the reconstructions presented here, have been deposited in the Electron Microscopy Public Image Archive (EMPIAR) with the associated codes EMPIAR-13121 (hPNPase^load-A, B^), EMPIAR-13120 (hPNPase^pre-cat^ and hPNPase^load-C^), and EMPIAR-13119 (hPNPase^apo-loop-conf1-3^).

## References

[1] Grunberg-Manago M, Oritz PJ, Ochoa S. Enzymatic synthesis of nucleic acidlike polynucleotides. Science. 1955;122:907–10. 10.1126/science.122.3176.907. 13274047

[2] Cheng ZF, Deutscher MP. Quality control of ribosomal RNA mediated by polynucleotide phosphorylase and RNase R. Proc Natl Acad Sci USA. 2003;100:6388–93. 10.1073/pnas.1231041100. 12743360 PMC164456

[3] Nurmohamed S, Vaidialingam B, Callaghan AJ et al. Crystal structure of *Escherichia coli* polynucleotide phosphorylase core bound to RNase E, RNA and manganese: implications for catalytic mechanism and RNA degradosome assembly. J Mol Biol. 2009;389:17–33. 10.1016/j.jmb.2009.03.051. 19327365 PMC2723993

[4] Wang DD, Shu Z, Lieser SA et al. Human mitochondrial SUV3 and polynucleotide phosphorylase form a 330-kDa heteropentamer to cooperatively degrade double-stranded RNA with a 3′-to-5′ directionality. J Biol Chem. 2009;284:20812–21. 10.1074/jbc.M109.009605. 19509288 PMC2742846

[5] Chen HW, Rainey RN, Balatoni CE et al. Mammalian polynucleotide phosphorylase is an intermembrane space RNase that maintains mitochondrial homeostasis. Mol Cell Biol. 2006;26:8475–87. 10.1128/MCB.01002-06. 16966381 PMC1636764

[6] Minczuk M, Piwowarski J, Papworth MA et al. Localisation of the human hSuv3p helicase in the mitochondrial matrix and its preferential unwinding of dsDNA. Nucleic Acids Res. 2002;30:5074–86. 10.1093/nar/gkf647. 12466530 PMC137961

[7] Dhir A, Dhir S, Borowski LS et al. Mitochondrial double-stranded RNA triggers antiviral signalling in humans. Nature. 2018;560:238–42. 10.1038/s41586-018-0363-0. 30046113 PMC6570621

[8] Silva S, Camino LP, Aguilera A. Human mitochondrial degradosome prevents harmful mitochondrial R loops and mitochondrial genome instability. Proc Natl Acad Sci USA. 2018;115:11024–9. 10.1073/pnas.1807258115. 30301808 PMC6205488

[9] Zhu X, Xie X, Das H et al. Non-coding 7S RNA inhibits transcription via mitochondrial RNA polymerase dimerization. Cell. 2022;185:2309–23. 10.1016/j.cell.2022.05.006. 35662414

[10] Matilainen S, Carroll CJ, Richter U et al. Defective mitochondrial RNA processing due to PNPT1 variants causes Leigh syndrome. Hum Mol Genet. 2017;26:3352–61. 10.1093/hmg/ddx221. 28645153

[11] Vedrenne V, Gowher A, De Lonlay P et al. Mutation in PNPT1, which encodes a polyribonucleotide nucleotidyltransferase, impairs RNA import into mitochondria and causes respiratory-chain deficiency. Am J Hum Genet. 2012;91:912–8. 10.1016/j.ajhg.2012.09.001. 23084291 PMC3487136

[12] von Ameln S, Wang G, Boulouiz R et al. A mutation in PNPT1, encoding mitochondrial-RNA-import protein PNPase, causes hereditary hearing loss. Am J Hum Genet. 2012;91:919–27. 10.1016/j.ajhg.2012.09.002. 23084290 PMC3487123

[13] Symmons MF, Jones GH, Luisi BF. A duplicated fold is the structural basis for polynucleotide phosphorylase catalytic activity, processivity, and regulation. Structure. 2000;8:1215–26. 10.1016/S0969-2126(00)00521-9. 11080643

[14] Dendooven T, Sinha D, Roeselová A et al. A cooperative PNPase-Hfq-RNA carrier complex facilitates bacterial riboregulation. Mol Cell. 2021;81:2901–13. 10.1016/j.molcel.2021.05.032. 34157309 PMC8294330

[15] Lin CL, Wang YT, Yang WZ et al. Crystal structure of human polynucleotide phosphorylase: insights into its domain function in RNA binding and degradation. Nucleic Acids Res. 2012;40:4146–57. 10.1093/nar/gkr1281. 22210891 PMC3351181

[16] Lorentzen E, Conti E. Crystal structure of a 9-subunit archaeal exosome in pre-catalytic states of the phosphorolytic reaction. Archaea. 2012;2012,721869. 23319881 10.1155/2012/721869 PMC3539426

[17] Büttner K, Wenig K, Hopfner KP. Structural framework for the mechanism of archaeal exosomes in RNA processing. Mol Cell. 2005;20:461–71. 16285927 10.1016/j.molcel.2005.10.018

[18] Deutscher MP, Reuven NB. Enzymatic basis for hydrolytic versus phosphorolytic mRNA degradation in *Escherichia coli* and *Bacillus subtilis*. Proc Natl Acad Sci USA. 1991;88:3277–80. 10.1073/pnas.88.8.3277. 1707536 PMC51429

[19] Wang W, Bechhofer DH. Properties of a *Bacillus subtilis* polynucleotide phosphorylase deletion strain. J Bacteriol. 1996;178:2375–82. 10.1128/jb.178.8.2375-2382.1996. 8636041 PMC177948

[20] Portnoy V, Palnizky G, Yehudai-Resheff S et al. Analysis of the human polynucleotide phosphorylase (PNPase) reveals differences in RNA binding and response to phosphate compared to its bacterial and chloroplast counterparts. RNA. 2008;14:297–309. 10.1261/rna.698108. 18083836 PMC2212259

[21] Li YC, Wang CH, Patra M et al. Structural insights into human PNPase in health and disease. Nucleic Acids Res. 2025;53:gkaf119. 39997218 10.1093/nar/gkaf119 PMC11851098

[22] Punjani A, Rubinstein JL, Fleet DJ et al. cryoSPARC: algorithms for rapid unsupervised cryo-EM structure determination. Nat Methods. 2017;14:290–6. 10.1038/nmeth.4169. 28165473

[23] Bepler T, Morin A, Rapp M et al. Positive-unlabeled convolutional neural networks for particle picking in cryo-electron micrographs. Nat Methods. 2019;16:1153–60. 10.1038/s41592-019-0575-8. 31591578 PMC6858545

[24] Kimanius D, Jamali K, Wilkinson ME et al. Data-driven regularization lowers the size barrier of cryo-EM structure determination. Nat Methods. 2024;21:1216–21. 10.1038/s41592-024-02304-8. 38862790 PMC11239489

[25] Zivanov J, Nakane T, Scheres SHW. Estimation of high-order aberrations and anisotropic magnification from cryo-EM data sets in. IUCrJ. 2020;7:253–67. 10.1107/S2052252520000081. PMC7055373 32148853

[26] Scheres SH . A Bayesian view on cryo-EM structure determination. J Mol Biol. 2012;415:406–18. 10.1016/j.jmb.2011.11.010. 22100448 PMC3314964

[27] Asarnow D, Palovcak E, Cheng Y. UCSF pyem v0.5 Zenodo. 2019. 10.5281/zenodo.3576630 (27 October 2025, date last accessed).

[28] Jumper J, Evans R, Pritzel A et al. Highly accurate protein structure prediction with AlphaFold. Nature. 2021;596:583–9. 10.1038/s41586-021-03819-2. 34265844 PMC8371605

[29] Croll TI . ISOLDE: a physically realistic environment for model building into low-resolution electron-density maps. Acta Crystallogr D Struct Biol. 2018;74:519–30. 10.1107/S2059798318002425. 29872003 PMC6096486

[30] Emsley P, Cowtan K. Coot: model-building tools for molecular graphics. Acta Crystallogr D Biol Crystallogr. 2004;60:2126–32. 10.1107/S0907444904019158. 15572765

[31] Liebschner D, Afonine PV, Baker ML et al. Macromolecular structure determination using X-rays, neutrons and electrons: recent developments in Phenix. Acta Crystallogr D Struct Biol. 2019;75:861–77. 10.1107/S2059798319011471. 31588918 PMC6778852

[32] Williams CJ, Headd JJ, Moriarty NW et al. MolProbity: more and better reference data for improved all-atom structure validation. Protein Sci. 2018;27:293–315. 10.1002/pro.3330. 29067766 PMC5734394

[33] Tan YZ, Baldwin PR, Davis JH et al. Addressing preferred specimen orientation in single-particle cryo-EM through tilting. Nat Methods. 2017;14:793–6. 10.1038/nmeth.4347. 28671674 PMC5533649

[34] Cardone G, Heymann JB, Steven AC. One number does not fit all: mapping local variations in resolution in cryo-EM reconstructions. J Struct Biol. 2013;184:226–36. 10.1016/j.jsb.2013.08.002. 23954653 PMC3837392

[35] Pettersen EF, Goddard TD, Huang CC et al. UCSF ChimeraX: structure visualization for researchers, educators, and developers. Protein Sci. 2021;30:70–82. 10.1002/pro.3943. 32881101 PMC7737788

[36] He J, Li T, Huang SY. Improvement of cryo-EM maps by simultaneous local and non-local deep learning. Nat Commun. 2023;14:3217. 10.1038/s41467-023-39031-1. 37270635 PMC10239474

[37] Pei J, Kim BH, Grishin NV. PROMALS3D: a tool for multiple protein sequence and structure alignments. Nucleic Acids Res. 2008;36:2295–300. 10.1093/nar/gkn072. 18287115 PMC2367709

[38] Robert X, Gouet P. Deciphering key features in protein structures with the new ENDscript server. Nucleic Acids Res. 2014;42:W320–4. 10.1093/nar/gku316. 24753421 PMC4086106

[39] Warnecke JM, Fürste JP, Hardt WD et al. Ribonuclease P (RNase P) RNA is converted to a Cd(2+)-ribozyme by a single Rp-phosphorothioate modification in the precursor tRNA at the RNase P cleavage site. Proc Natl Acad Sci USA. 1996;93:8924–8. 10.1073/pnas.93.17.8924. 8799129 PMC38570

[40] Teramoto T, Koyasu T, Yokogawa T et al. Structural basis of transfer RNA processing by bacterial minimal RNase P. Nat Commun. 2025;16:5456. 10.1038/s41467-025-60002-1. 40593470 PMC12217051

[41] Miller LG, Kim W, Schowe S et al. Selective 8-oxo-rG stalling occurs in the catalytic core of polynucleotide phosphorylase (PNPase) during degradation. Proc Natl Acad Sci USA. 2024;121:e2317865121. 10.1073/pnas.2317865121. 39495922 PMC11572968

[42] Unciuleac MC, Shuman S. Discrimination of RNA from DNA by polynucleotide phosphorylase. Biochemistry. 2013;52:6702–11. 10.1021/bi401041v. 23980617 PMC3791318

[43] Mohanty BK, Kushner SR. Polynucleotide phosphorylase functions both as a 3′ right-arrow 5′ exonuclease and a poly(A) polymerase in *Escherichia coli*. Proc Natl Acad Sci USA. 2000;97:11966–71. 10.1073/pnas.220295997. 11035800 PMC17278

[44] Shadel GS, Horvath TL. Mitochondrial ROS signaling in organismal homeostasis. Cell. 2015;163:560–9. 10.1016/j.cell.2015.10.001. 26496603 PMC4634671

[45] Wang G, Chen HW, Oktay Y et al. PNPASE regulates RNA import into mitochondria. Cell. 2010;142:456–67. 10.1016/j.cell.2010.06.035. 20691904 PMC2921675

[46] Borowski LS, Dziembowski A, Hejnowicz MS et al. Human mitochondrial RNA decay mediated by PNPase-hSuv3 complex takes place in distinct foci. Nucleic Acids Res. 2013;41:1223–40. 10.1093/nar/gks1130. 23221631 PMC3553951

