# Supplementary material for: Loop-mediated regulation and base flipping drive RNA cleavage by human mitochondrial PNPase
Source: Nucleic Acids Res. 2025 Dec 9;53(22):gkaf1296. doi: 10.1093/nar/gkaf1296 (PMC12685370; doi:10.1093/nar/gkaf1296)
Supplement: gkaf1296_Supplemental_File [file gkaf1296_supplemental_file.pdf]

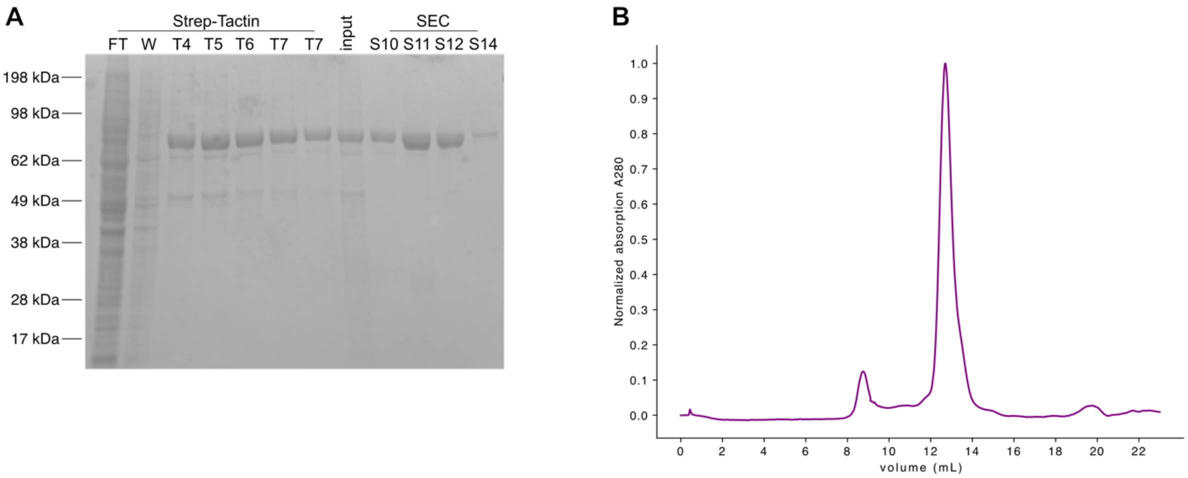

Supplementary Figure 1: hPNPase protein purification using Strep-Tactin XT 4Flow affinity purification (IBA) and size exclusion chromatography (SEC). (**A**) Coomassie-stained 4-12 % SDS-PAGE (Invitrogen) showing protein ladder (Invitrogen SeeBlue™ Plus2 Pre-stained Protein Standard), Strep-Tactin flowthrough (FT), wash (W) and elution (T) fractions, input for SEC and SEC elution fraction (S). (**B**) SEC chromatogram of hPNPase, the normalized intensity at A280 is plotted against the elution volume, and a Superdex 200 10/300 GL column (Cytiva) was used.

Supplementary Table 1 Oligoribonucleotides and Oligonucleotides used in this study.

Name	Sequence	Application
FAM_PNP_RNA_long	/56-FAM/rCrArC rArArC rArCrA rCrArC rCrArC rArCrA rCrArC rArUrA rArArA rCrArA rArArC rArGrC rUrArC rGrCrC rArUrC rCrUrC rCrCrC rCrCrA rArUrC rUrA	RNA degradation assay, RNA/DNA competition assay, cryo-EM, mass spectrometry
FAM_PNP_RNA_PT5_long	/56-FAM/rCrArCrArArCrArCrArCrArCrCrArCrArCrArCrArCrArUrArArA rArCrArArArArCrArGrCrUrArCrGrCrCrArUrC*rC*rU*rC*rC*rCrCrCr CrArArUrCrUrA	RNA degradation assay, mass spectrometry
FAM_PNP_RNA_PT10_long	/56-FAM/rCrA rCrArA rCrArC rArCrA rCrCrA rCrArC rArCrA rCrArU rArArA rArCrA rArArA rCrArG rCrUrA rCrG*rC* rC*rA*rU* rC*rC*rU* rC*rC*rC rCrCrC rArArU rCrUrA	RNA degradation assay, mass spectrometry
FAM_PNP_RNA_OMe10_lon g	/56-FAM/rCrA rCrArA rCrArC rArCrA rCrCrA rCrArC rArCrA rCrArU rArArA rArCrA rArArA rCrArG rCrUrA rCrGC mCmAmU mCmCmU mCmCmC rCrCrC rArArU rCrUrA	RNA degradation assay, mass spectrometry
FAM_PNP_RNA_PT1_short	/56-FAM/rArC rArCrA rArCrA rCrArA rCrArC rArCrA rCrCrA rCrArC rArCrA rCrArU rArArA rArCrA rArArA rC*rA	RNA degradation assay
FAM_PNP_RNA_PT20_short	/56-FAM/rArC rArCrA rArCrA rCrArA rCrArC rArCrA rCrCrA* rCrArC rArCrA rCrArU rArArA rArCrA rArArA rCrA	RNA degradation assay
FAM_PNP_RNA_PT21_short	/56-FAM/rArC rArCrA rArCrA rCrArA rCrArC rArCrA rCrC*rA rCrArC rArCrA rCrArU rArArA rArCrA rArArA rCrA	RNA degradation assay
FAM_PNP_RNA_3`-PO3_short	/56-FAM/rArCrArCrArArCrArC rArArC rArCrArCrArCrCrArC rArCrA rCrArCrArUrArArArA rCrArA rArArC rA PO3	RNA degradation assay
FAM_3x_rG	/56-FAM/rGrArCrGrArArArCrArGrGrGrCrUrArArArGrArU	RNA degradation assay
FAM_3x_oxo_rG	/56-FAM/rGrArCrGrArArArCrA/i8oxorG/i8oxorG/i8oxorG/rCrUrArArA rGrArU	RNA degradation assay
FAM_PNP_DNA_short	/56-FAM/AC ACA ACA CAA CAC ACA CCA CAC ACA CAT AAA ACA AAA CA	DNA degradation assay
PNP_DNA_long	CAC AAC ACA CAC CAC ACA CAC ATA AAA CAA AAC AGC TAC GCC ATC CTC CCC CCA ATC TA	RNA/DNA competition assay
PNP_RNA_PT5_short	rArCrArCrArArCrArC rArArC rArCrArCrArCrCrArC rArCrA rCrA*rC* rA*rU*rA* rArArA rCrArA rArArC rA	Cryo-EM
DNA-RNA_control	5´-CCACCACACACACATAAACrArArArArCrA-3`	RNA degradation assay
DNA-RNA-PT5_pos2	5´-CCACCACACACACATAAAC*rA*rA*rA*rArC*rA-3`	RNA degradation assay

“56-FAM” indicate FAM labelling, “*” phosphorothioate modification, “m” 2`-OH methylation, and “i8oxorG” 8-hydroxyguanosine (8-oxo)-rG. Each of them was synthesized by IDT

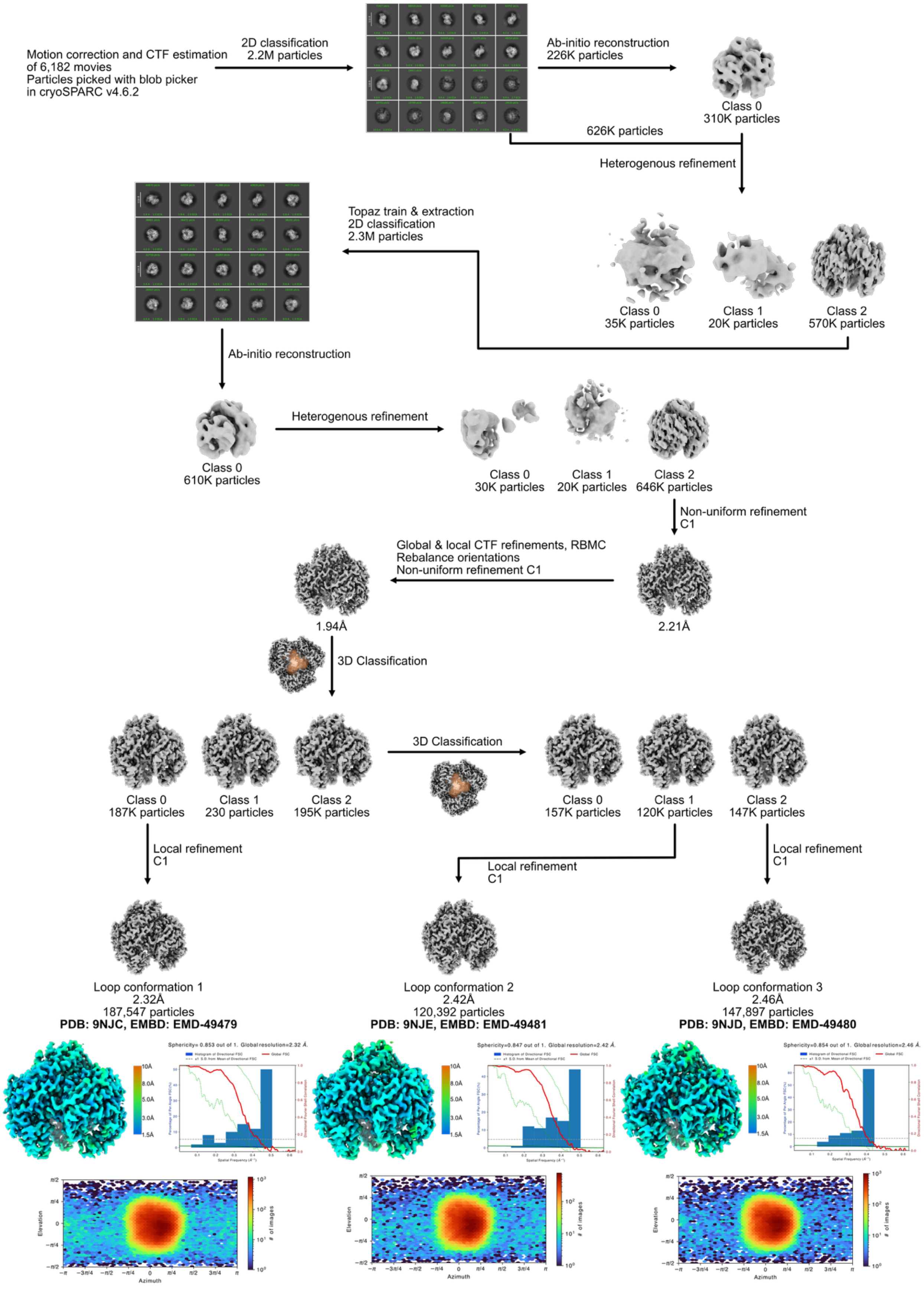
Motion correction and CTF estimation
of 6,182 movies
Particles picked with blob picker
in cryoSPARC v4.6.2
2D classification
2.2M particles
Ab-initio reconstruction
226K particles
Class 0
310K particles
626K particles
Heterogenous refinement
Topaz train & extraction
2D classification
2.3M particles
Class 0
35K particles
Class 1
20K particles
Class 2
570K particles
Ab-initio reconstruction
Class 0
610K particles
Heterogenous refinement
Class 0
30K particles
Class 1
20K particles
Class 2
646K particles
Non-uniform refinement
C1
Global & local CTF refinements, RBMC
Rebalance orientations
Non-uniform refinement C1
1.94Å
2.21Å
3D Classification
Class 0
187K particles
Class 1
230 particles
Class 2
195K particles
3D Classification
Class 0
157K particles
Class 1
120K particles
Class 2
147K particles
Local refinement
C1
Local refinement
C1
Local refinement
C1
Loop conformation 1
2.32Å
187,547 particles
PDB: 9NJC, EMBD: EMD-49479
Loop conformation 2
2.42Å
120,392 particles
PDB: 9NJE, EMBD: EMD-49481
Loop conformation 3
2.46Å
147,897 particles
PDB: 9NJD, EMBD: EMD-49480
Sphericity= 0.853 out of 1. Global resolution=2.32 Å.
Sphericity=0.847 out of 1. Global resolution=2.42 Å.
Sphericity=0.854 out of 1. Global resolution=2.46 Å.
10Å
8.0Å
5.0Å
3.0Å
1.5Å
Elevation
Azimuth
# of images

Supplementary Figure 2: hPNPase bound to Pi in loop conformation 1 (hPNPase$^{apo-loop-conf1}$; PDB code: 9NJC), 2 (hPNPase$^{apo-loop-conf2}$; PDB code: 9NJE), and 3 (hPNPase$^{apo-loop-conf3}$; PDB code: 9NJD) cryo-EM processing scheme. Motion correction and CTF estimation were performed in cryoSPARC v4.6.2 (22). Particle picking was initially accomplished with blob picker, followed by Topaz training and extraction (23). Cryo-EM reconstruction evaluation plots. The local resolutions were calculated using blocRES (34). The 3D Fourier Shell Correlations (FSCs) of the final local (trimeric PNPase focus) refinements with 3DFSC (33), and angular orientations of particles used in the final refinements with cryoSPARC v4.6.2 (22). The masks for 3DFSC calculation were generated in CryoSPARC v4.6.2 (22) using a relative threshold of 0.5.

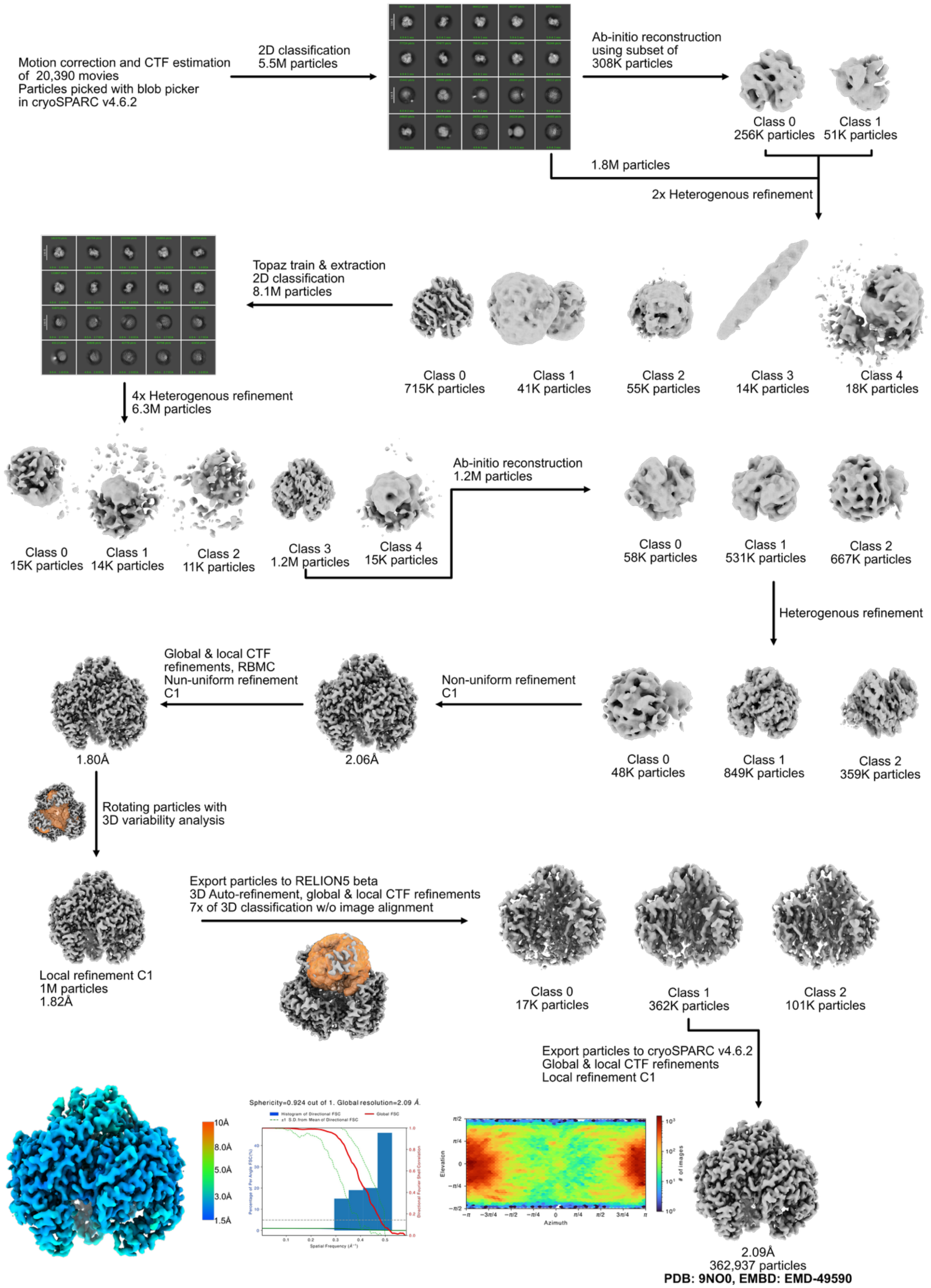
Motion correction and CTF estimation of 20,390 movies
Particles picked with blob picker in cryoSPARC v4.6.2
2D classification
5.5M particles
Ab-initio reconstruction using subset of 308K particles
Class 0
256K particles
Class 1
51K particles
1.8M particles
2x Heterogenous refinement
Class 0
715K particles
Class 1
41K particles
Class 2
55K particles
Class 3
14K particles
Class 4
18K particles
Topaz train & extraction
2D classification
8.1M particles
4x Heterogenous refinement
6.3M particles
Class 0
15K particles
Class 1
14K particles
Class 2
11K particles
Class 3
1.2M particles
Class 4
15K particles
Ab-initio reconstruction
1.2M particles
Class 0
58K particles
Class 1
531K particles
Class 2
667K particles
Heterogenous refinement
Class 0
48K particles
Class 1
849K particles
Class 2
359K particles
Non-uniform refinement
C1
2.06Å
Global & local CTF refinements, RBMC
Nun-uniform refinement
C1
1.80Å
Rotating particles with 3D variability analysis
Local refinement C1
1M particles
1.82Å
Export particles to RELION5 beta
3D Auto-refinement, global & local CTF refinements
7x of 3D classification w/o image alignment
Class 0
17K particles
Class 1
362K particles
Class 2
101K particles
Export particles to cryoSPARC v4.6.2
Global & local CTF refinements
Local refinement C1
10Å
8.0Å
5.0Å
3.0Å
1.5Å
Sphericity=0.924 out of 1. Global resolution=2.09 Å.
Spatial Frequency
Elevation
Azimuth
2.09Å
362,937 particles
PDB: 9NO0, EMBD: EMD-49590

Supplementary Figure 3: hPNPase bound to RNA in loading state (hPNPase^{load}; PDB code: 9NO0) cryo-EM processing scheme. Motion correction and CTF estimation were performed in cryoSPARC v4.6.2 (22). Particle picking was initially accomplished with a blob picker, followed by Topaz training and extraction (23). All steps were performed in cryoSPARC v4.6.2 (22), except for 3D classification on the RNA-containing protomer, performed in RELION5-beta (24-26) without image alignments. Cryo-EM reconstruction evaluation plots. The local resolution was calculated using blocRES (34). The 3D Fourier Shell Correlation (FSC) of the final local (trimeric PNPase focus) refinements with 3DFSC (33), and angular orientations of particles used in the final refinement with cryoSPARC v4.6.2 (22). The mask for 3DFSC calculation was generated in CryoSPARC v4.6.2 (22) using a relative threshold of 0.5.

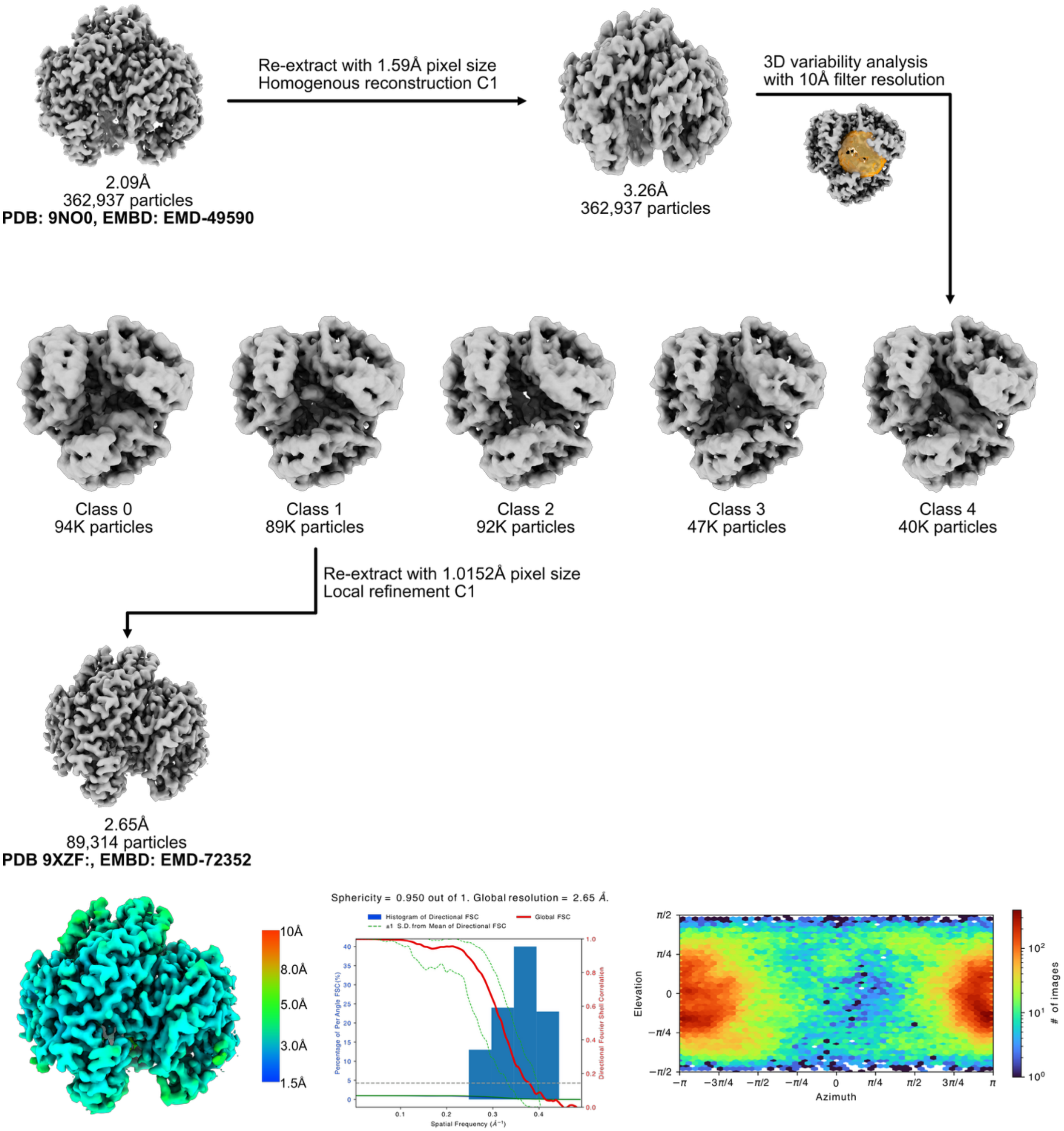

Supplementary Figure 4: hPNPase bound to RNA in loading state B (hPNPase$^{load\text{-}B}$; PDB code: 9XZF) cryo-EM processing scheme. The final particles for hPNPase bound to RNA in loading state (hPNPase^{load}, Supplementary Figure 3) were further classified using a focused mask and 3D variability analysis. All steps were performed in cryoSPARC v4.7.1 (22). Cryo-EM reconstruction evaluation plots. The local resolution was calculated using blocRES (34). The 3D Fourier Shell Correlation (FSC) of the final local (trimeric PNPase focus) refinements with 3DFSC (33), and angular orientations of particles used in the final refinement with cryoSPARC v4.7.1 (22). The mask for 3DFSC calculation was generated in CryoSPARC v4.7.1 (22) using a relative threshold of 0.5.

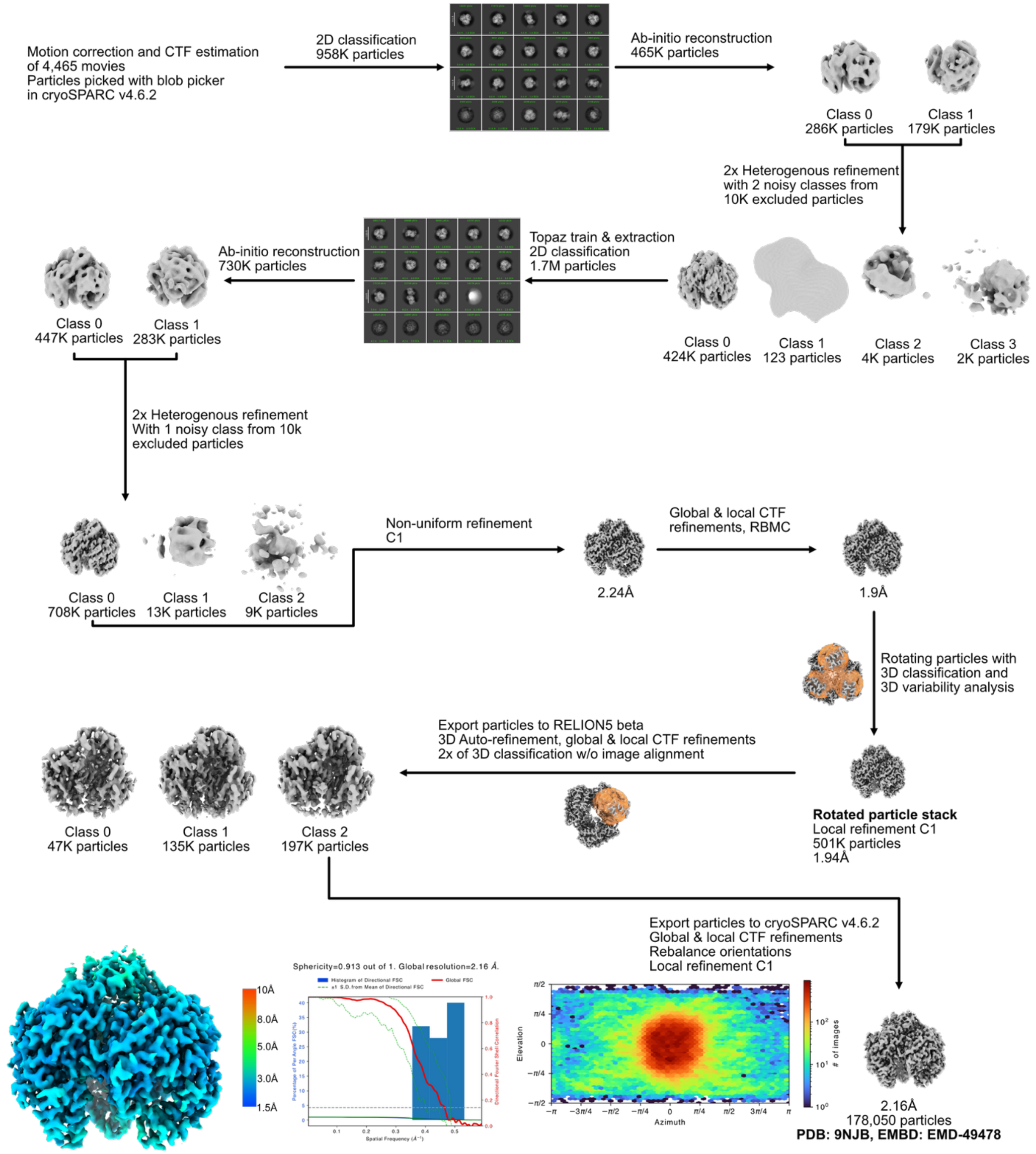

Supplementary Figure 5: hPNPase bound to RNA in pre-catalytic state (hPNPase$^{pre\text{-}cat}$; PDB code: 9NJB) cryo-EM processing scheme. Motion correction and CTF estimation were performed in cryoSPARC v4.6.2 (22). Particle picking was initially performed with blob picker, followed by Topaz training and extraction (23). All steps were performed in cryoSPARC v4.6.2 (22) except for 3D classification on the RNA-containing protomer, performed in RELION5-beta (24-26) without image alignments. Cryo-EM reconstruction evaluation plots. The local resolution was calculated using blocRES (34). The 3D Fourier Shell Correlation (FSC) of the final local (trimeric PNPase focus) refinements with 3DFSC (33), and angular orientations of particles used in the final refinement with cryoSPARC v4.6.2 (22). The mask for 3DFSC calculation was generated in CryoSPARC v4.6.2 (22) using a relative threshold of 0.5.

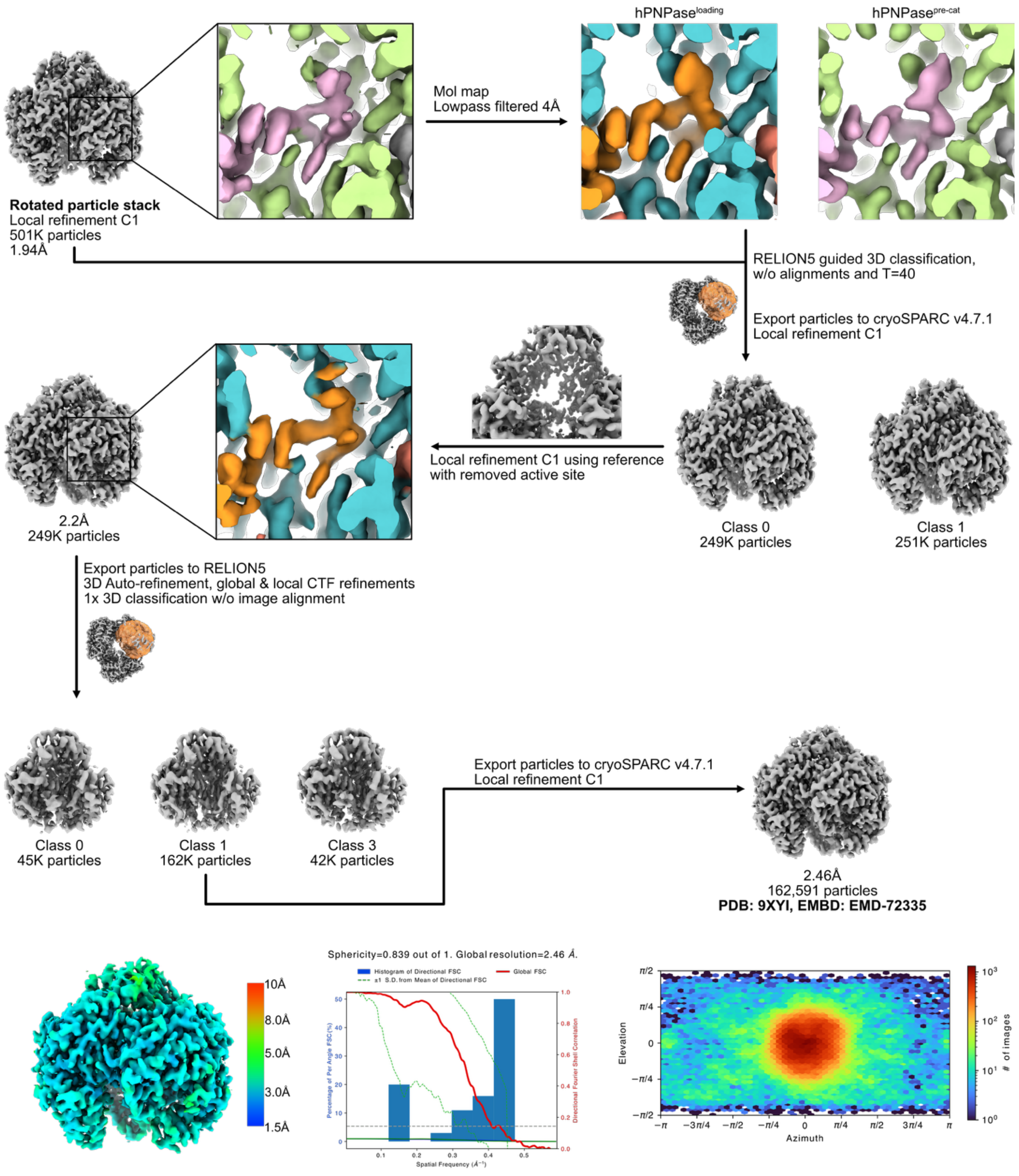

Supplementary Figure 6: hPNPase bound to RNA in loading state C (hPNPase$^{load\text{-}C}$; PDB code: 9XYI) cryo-EM processing scheme. After alignment of the RNA containing hPNPase particles (rotated particle stack, Supplementary Figure 5), the reconstruction of the RNA resembled a heterogeneous state consisting of hPNPase in loading and pre-catalytic state. Molmaps of hPNPase in loading and pre-catalytic conformation were generated in ChimeraX (35) and used for guided 3D classification without image alignments in RELION5 (24-26). The classified particles were exported to cryoSPARC v4.7.1 (22), and a local refinement was performed using an input volume lacking the active site to avoid model bias. Further non-guided 3D

classification was performed in RELION5 (24,26), and the final particles were refined in cryoSPARC v4.7.1 (22). Cryo-EM reconstruction evaluation plots. The local resolution was calculated using blocRES (34). The 3D Fourier Shell Correlation (FSC) of the final local (trimeric PNPase focus) refinements with 3DFSC (33), and angular orientations of particles used in the final refinement with cryoSPARC v4.6.2 (22). The mask for 3DFSC calculation was generated in CryoSPARC v4.6.2 (22) using a relative threshold of 0.5.

Supplementary Table 2 Cryo-EM data collection, refinement, and validation statistics.

	**hPNPase** load-A	**hPNPase** load-B	**hPNPase** pre-cat	**hPNPase** load-C	**hPNPase** apo-loop-conf1	**hPNPase** apo-loop-conf2	**hPNPase** apo-loop-conf3
EMDB code	EMD-49590	EMD-72352	EMD-49478	EMD-72335	EMD-49479	EMD-49481	EMD-49480
PDB code	9NO0	9XZF	9NJB	9XYI	9NJC	9NJE	9NJD
**Data collection and processing**							
Nominal EF-TEM Magnification	165,000		165,000		165,000		
Voltage (kV), source	300, E-CFEG		300, E-CFEG		300, E-CFEG		
Electron exposure ($e^-/Å^2$)	58.4/59.8		48.6		45.98		
Underfocus range (μM)	0.3 to 2		0.3 to 1.5		0.3 to 1.5		
Pixel size (Å)	0.5076		0.5076		0.5076		
Symmetry imposed	C1		C1		C1		
Micrographs (no.)	20,390		4,465		6,182		
Initial particle images (no.)	8,230,572		1,800,445		2,237,191		
Final particle images (no.)	362,937	89,314	178,050	162,591	187,547	120,392	147,897
Map resolution (Å)[a]	2.08	2.65	2.15	2.46	2.36	2.44	2.44
FSC threshold	0.143	0.143	0.143	0.143	0.143	0.143	0.143

**Map resolution range (Å)[b]**							
Min,25th percentile	1.915, 2.228	2.286, 2.810	1.867, 2.367	2.003, 2.690	1.730, 2.635	1.883, 2.630	1.730, 2.698
Median, 75th percentile	2.595, 4.385	3.219, 5.305	2.722, 4.377	3.099, 5.355	2.996, 5.356	3.056, 5.412	3.164, 5.634
Max	29.493	36.062	35.254	39.938	37.760	37.815	38.012
**Structure refinement**							
Initial model used (Alphafold)	Q8TCS8	Q8TCS8	Q8TCS8	Q8TCS8	Q8TCS8	Q8TCS8	Q8TCS8
Model resolution (Å)	2.2	2.8	2.4	2.6	2.5	2.5	2.6
FSC threshold	0.5	0.5	0.5	0.5	0.5	0.5	0.5
**Model composition**							
Non-hydrogen atoms	14498	14563	14410	14496	14373	14245	14362
Protein/RNA residues	1871/6	1871/9	1861/6	1871/6	1871/0	1855/0	1869
Ligands	PO4: 3	PO4: 3	SO4: 3, MG: 2	SO4: 3	PO4: 3	PO4: 3	PO4: 3
**B factors (Å^2)**							
Protein (chain A/B/C)	41.40/176.40/ 80.27	76.65/218.41/ 118.46	37.27/166.04/ 79.01	46.74/173.01/ 87.65	42.86/170.02/ 79.62	38.96/171.21/ 77.96	41.44/170.84/ 76.88
RNA (chain D)	93.71/139.87/ 118.47	140.94/260.71/ 193.56	90.41/154.60/ 121.00	97.68/150.76/ 126.99	-	-	-
Ligands (chain A/B/C)	75.33/100.40/ 86.70	117.55/148.50/ 132.51	87.20/117.48/ 98.41	94.63/125.52/ 108.19	74.70/90.74/ 82.67	69.25/96.18/ 80.28	67.37/96.76/ 78.50

**r.m.s. deviations**							
Bond lengths (Å)	0.005	0.004	0.003	0.002	0.004	0.006	0.003
Bond angles (°)	0.550	0.467	0.503	0.467	0.487	0.574	0.471
**Validation**							
MolProbity score	1.32	1.35	1.35	1.41	1.60	1.76	1.46
Clashscore[c]	3.97	4.47	4.55	4.66	4.34	5.46	5.21
Rotamer outliers (%)	1.51	1.45	1.46	1.57	2.83	1.91	1.89
**Ramachandran plot[d]**							
Favored (%)	98.07	98.02	98.49	97.91	97.80	96.75	97.96
Allowed (%)	1.93	1.98	1.57	2.09	2.20	3.25	2.04
Disallowed (%)	0.00	0.00	0.00	0.00	0.00	0.00	0.00

[a]Gold-standard 0.143 FSC resolution calculated with 3DFSC (33). The masks for FSC calculation were generated in CryoSPARC v4.6.2 (22) using a relative threshold of 0.5. [b]Map resolution range calculated with CryoSPARC v4.6.2 (22) BlocRes (34) and FSC threshold of 0.5. [c]Clashscore calculated with MolProbity (32). [d]Ramachandran plot and outliers calculated with MolProbity (32).

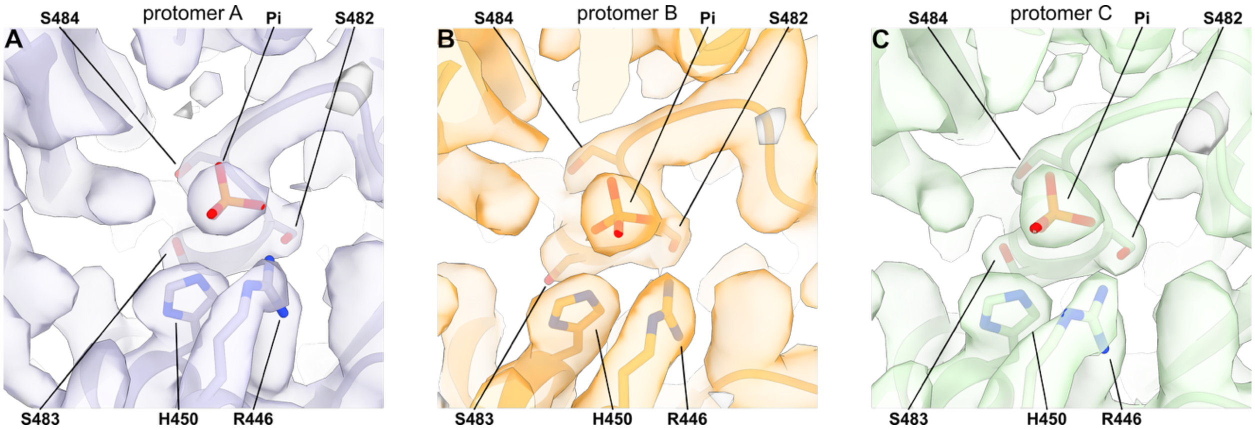

Supplementary Figure 7: hPNPase apo phosphate (Pi) map overlay. Semi-transparent Coulomb potential map (σ-level 0.0477) and model focused on the Pi binding site of hPNPase$^{apo\text{-}loop\text{-}conf1}$ (PDB code: 9NJC), in cartoon representation for (**A**) protomer A colored in blue, (**B**) protomer B colored in orange, and (**C**) protomer C colored in green. Amino acid side chains important for Pi binding and Pi are highlighted.

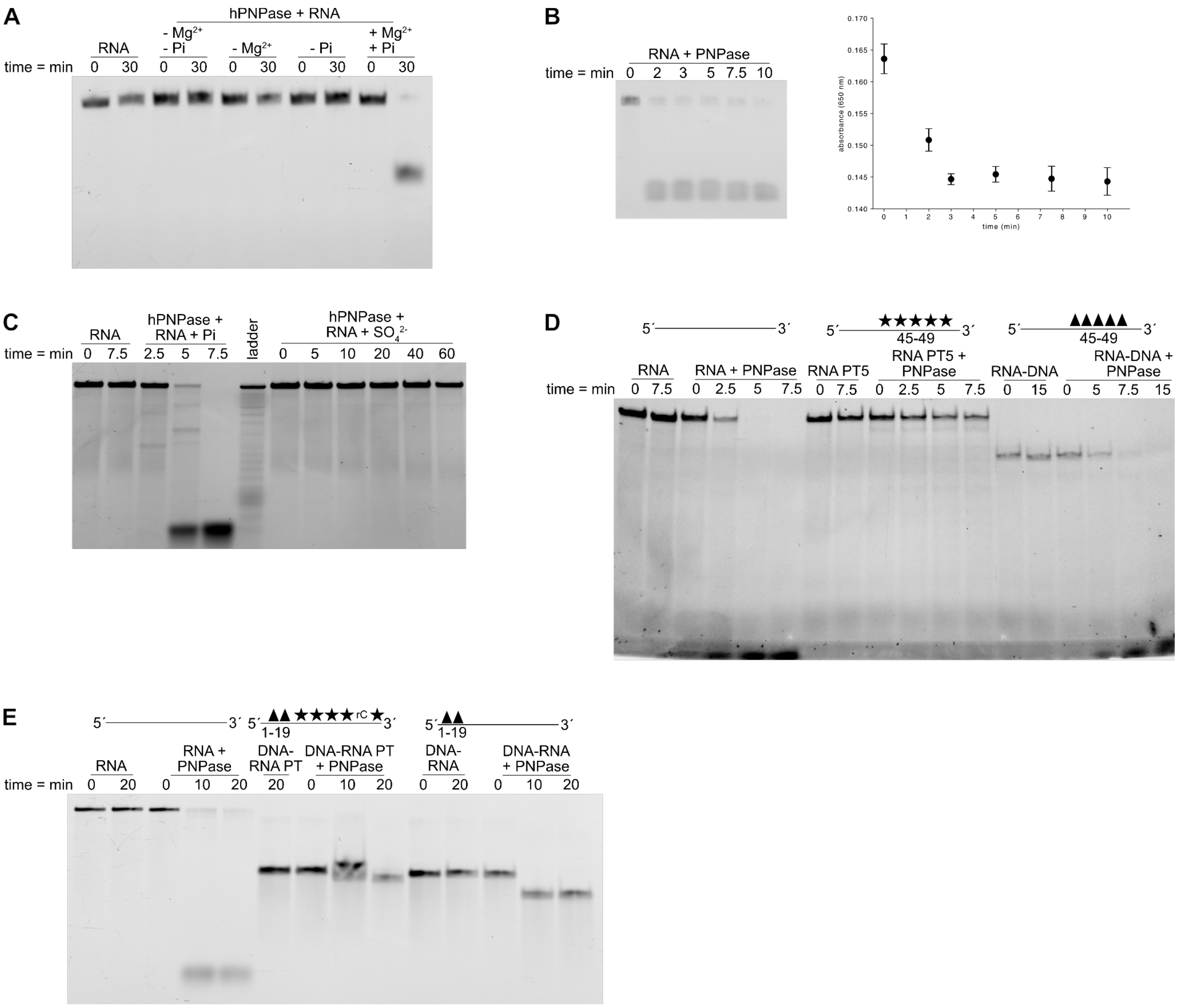

Supplementary Figure 8: Biochemical characterization of hPNPase with RNA degradation assays. (**A**) RNA degradation assay: Both Mg^{2+} and Pi are required for RNA degradation by hPNPase. (**B**) The time course of consumed Pi by hPNPase during RNA degradation was assessed with a malachite green assay. Each data point contains 3 replicates. A 15% TBE-Urea PAGE at similar time points was performed to validate the RNA degradation. (**C**) RNA degradation assay: hPNPase cannot degrade RNA when 2 mM Na_2SO_4 was used instead of NaH_2PO_4. (**D**) Five times phosphorothioate (PT5) modified RNA and five times DNA containing RNA (RNA-DNA) degradation assay: PT5 modified RNA and RNA-DNA reduced hPNPase degradation efficiency. (**E**) Five times phosphorothioate-modified (PT) RNA substrate interspaced by a single ribonucleotide in position 2 relative to the 3´-end degradation assay: PT modification does not significantly alter hPNPase RNA degradation behaviour. The RNAs, RNA modifications, and positions of the modifications are indicated for each sample. The phosphorothioate modifications are indicated as a star, DNA as a triangle, and ribonucleotides in hybrid oligonucleotides as "r". All RNAs contained a 5´-FAM label (Supplementary Table 1) and were resolved using 15% TBE-Urea PAGE (BioRad or Invitrogen).

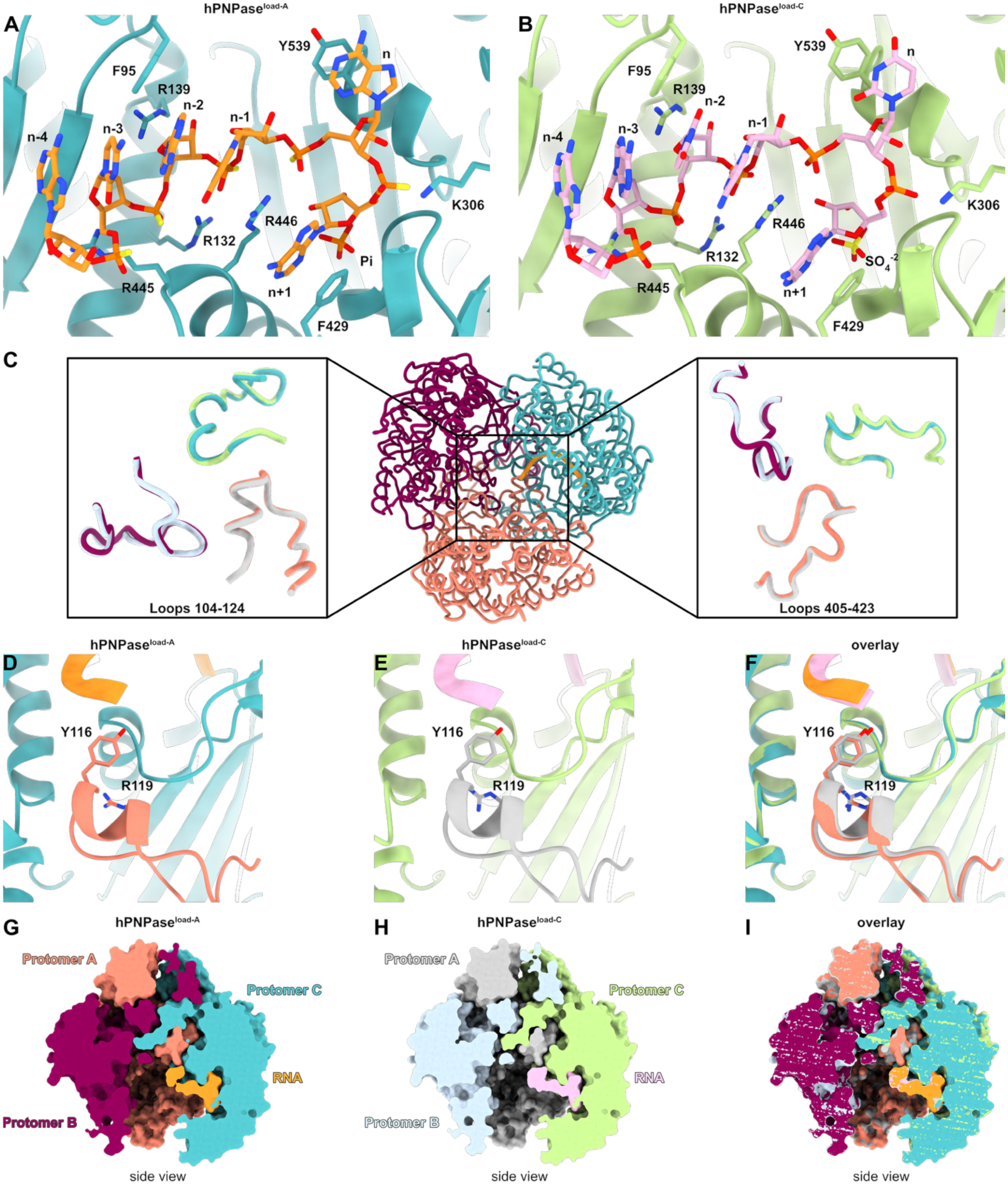

Supplementary Figure 9: Structural comparison between hPNPase in loading state derived from phosphorothioate-modified RNA cryo-EM dataset (hPNPase^{load-A}; PDB code: 9NO0; Supplementary Figure 3) and non-modified RNA cryo-EM dataset (hPNPase^{load-C}; PDB code: 9XYI; Supplementary Figure 6). Cartoon representation of trimeric hPNPase bound to RNA and co-substrate Pi in the active site in (**A**) hPNPase^{load-A} or (**B**) hPNPase^{load-C}. For RNA interaction, important residues and the co-substrate Pi are highlighted. (**C**) Close-up view of the pore loops spanning PH1 (residue 104-124) and PH2 (residue 405-

423) domains of each protomer for trimeric hPNPase bound to RNA in hPNPase$^{load\text{-}A}$ and hPNPase$^{Load\text{-}C}$ structures in cartoon representation is shown as an overlay. Each protomer is colored individually in violet, blue, and red for hPNPase$^{load\text{-}A}$ and green, gray, and light blue for hPNPase$^{load\text{-}C}$. Interaction between loops 104-124 with the adjacent active site for (**D**) hPNPase$^{load\text{-}A}$, (**E**) hPNPase$^{Load\text{-}C}$, and (**F**) an overlay shown as a cartoon representation. Residues important for the interaction between the loop and active site are shown. Side view surface representation of trimeric RNA-bound hPNPase in (**G**) hPNPase$^{load\text{-}A}$, (**H**) hPNPase$^{load\text{-}C}$, and (**I**) an overlay. Each protomer is colored individually in violet, blue, red, and RNA in orange for hPNPase$^{load\text{-}A}$ and light blue, green, gray, and RNA in pink for hPNPase$^{load\text{-}C}$, respectively.

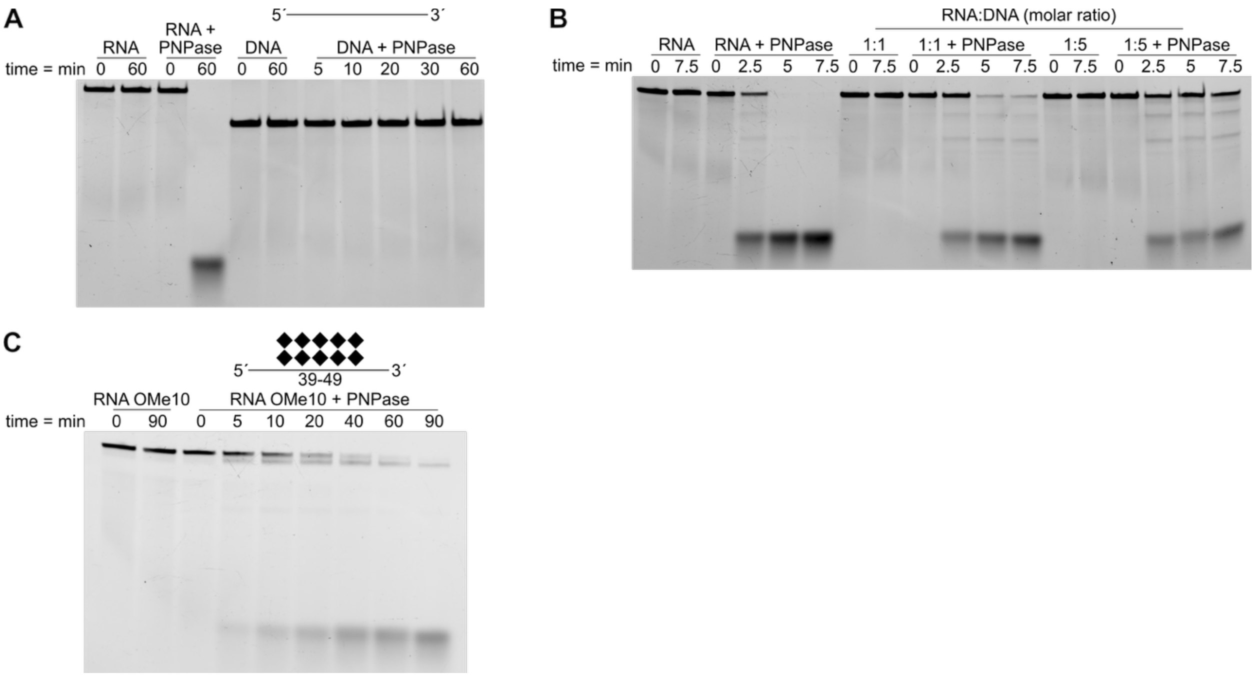

Supplementary Figure 10: Biochemical characterization of hPNPase with RNA degradation assays. (**A**) DNA degradation assay: hPNPase cannot degrade ssDNA when Pi and Mg^{2+} are present. (**B**) RNA-DNA competition assay: Increasing DNA concentrations decreased hPNPase RNA degradation efficiency. (**C**) 2´-OMe (OMe10) modified RNA degradation assay: OMe10 modification reduced hPNPase degradation efficiency. The RNAs or DNA, RNA modifications, and positions of the modifications are indicated for each sample. The 2´-Ome modification is indicated as a rectangle with black filling. All RNAs and DNA used in the degradation assay, except for the competing DNA in (**B**), contained a 5´-FAM label (Supplementary Table 1) and were resolved using 15% TBE-Urea PAGE (BioRad or Invitrogen).

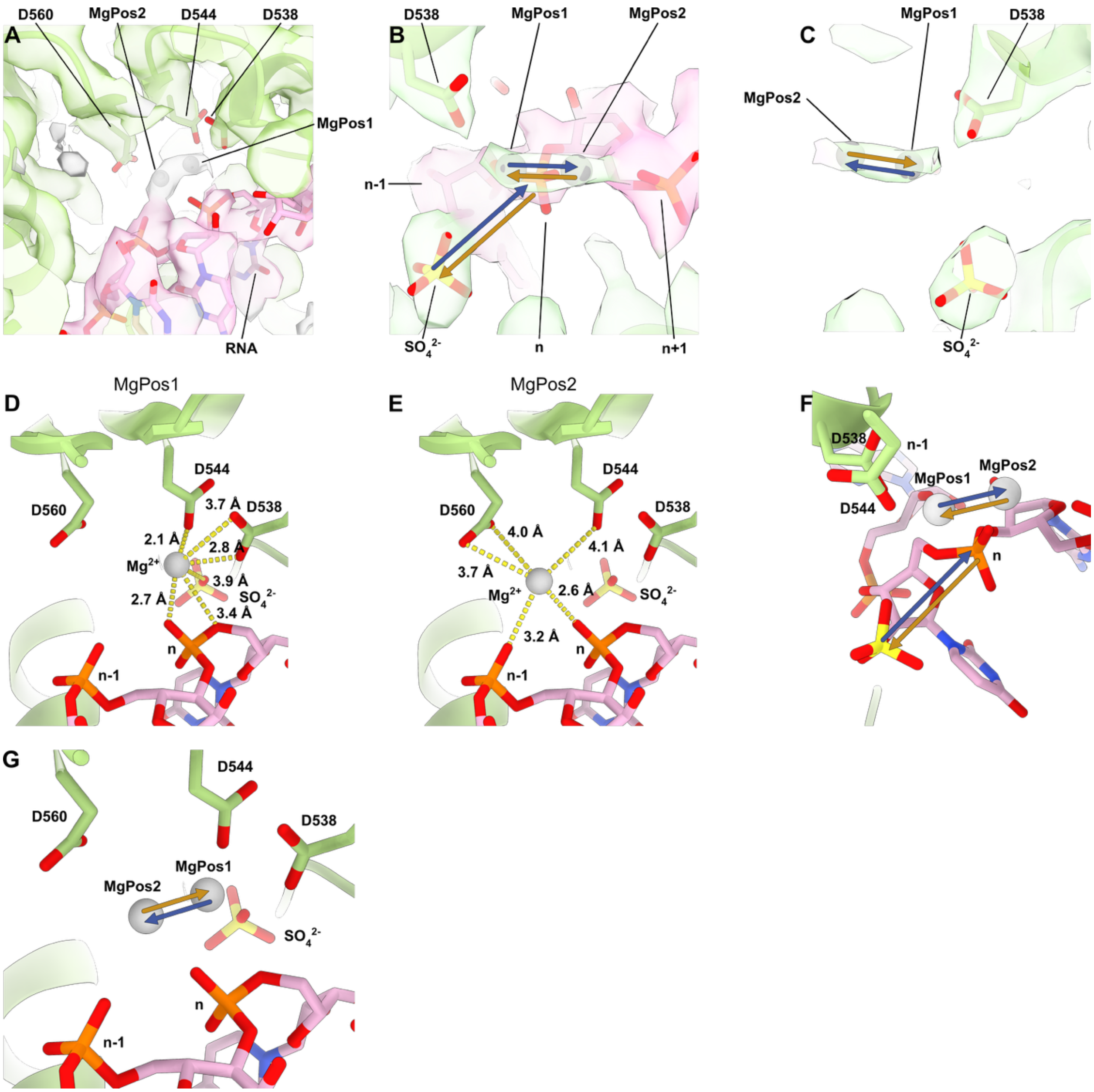

Supplementary Figure 11: Magnesium map overlay and binding of RNA-bound trimeric hPNPase in pre-catalytic state (hPNPase$^{pre\text{-}cat}$; PDB code: 9NJB). (**A**) Semi-transparent Coulomb potential map (σ-level 0.0458) overlayed with an atomic model of hPNPase$^{pre\text{-}cat}$ in cartoon representation. RNA, magnesium ion (Mg^{2+}) in position 1 (MgPos1) or position 2 (MgPos2), and important residues are highlighted in pink, gray, and green. (**B, C**) Semi-transparent cryo-EM Coulomb potential map (σ-level 0.0458) overlayed with a cartoon representation of hPNPase$^{pre\text{-}cat}$ focused on the magnesium-ion Coulomb potential map overlay. RNA, magnesium-ion (Mg^{2+}) in position 1 (MgPos1) or position 2 (MgPos2), and important residues are highlighted in pink, gray, and green. The dynamic movement of the magnesium ion upon nucleophilic attack of the Pi (here SO_4^{2-}) is indicated with arrows. Once Pi (here SO_4^{2-}) attacks in the RNA scissile bond, the magnesium ion is forced into the less favoured position 2 (MgPos2). (**D**) MgPos1 and (**E**) MgPos2. RNA, magnesium-ion (Mg^{2+}) in position 1 (MgPos1) or position 2 (MgPos2), and important residues are highlighted in pink, grey, and green, and interactions are indicated as dashed lines. (**F, G**) Cartoon

representation of $hPNPase^{pre\text{-}cat}$ focused on the magnesium ion. RNA, magnesium-ion (Mg^{2+}) in position 1 (MgPos1) or position 2 (MgPos2), and important residues are highlighted in pink, gray, and green. The dynamic movement of the magnesium ion upon nucleophilic attack of the Pi (here SO_4^{2-}) is indicated with arrows. Once Pi (here SO_4^{2-}) attacks the RNA scissile bond, the magnesium ion is forced into the less favoured position 2 (MgPos2).

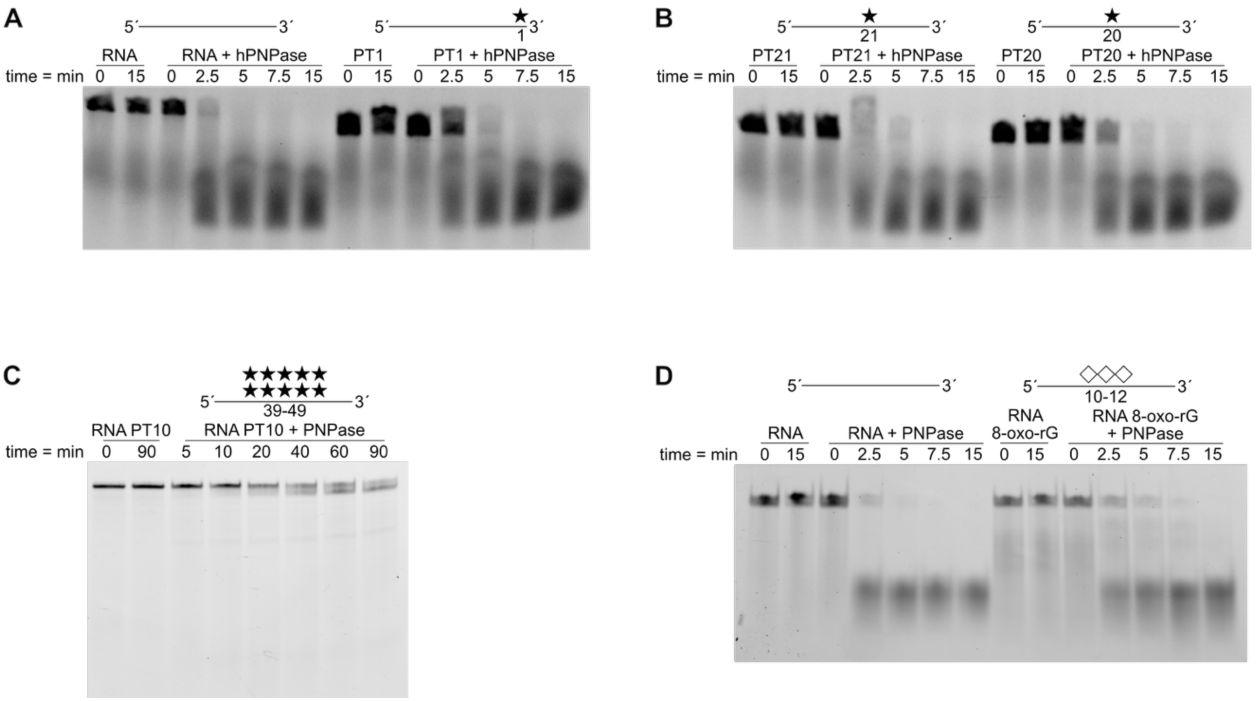

Supplementary Figure 12: Biochemical characterization of hPNPase with RNA degradation assays. (**A**) PT-modified RNA in position 1 relative to the 3′-end RNA degradation assay: hPNPase degraded the substrate without decreased efficiency. (**B**) No cleavable PT-modified RNAs in positions 20 and 21 relative to the 3′-end RNA degradation assay: hPNPase degraded all substrates without decreased efficiency. (**C**) Ten times Phosphorothioate (PT10) modified RNA degradation assay: PT10 modified RNA reduced hPNPase degradation efficiency. (**D**) Three times 8-oxo-rG modified RNA degradation assay: 8-oxo-rG modified RNA only slightly decreases degradation efficiency. The RNAs, RNA modifications, and positions of the modifications are indicated for each sample. The phosphorothioate modifications are indicated as a star, and 8-oxo-rG as a rectangle with white filling. All RNAs contained a 5′-FAM label (Supplementary Table 1) and were resolved using 15% TBE-Urea PAGE (BioRad or Invitrogen).

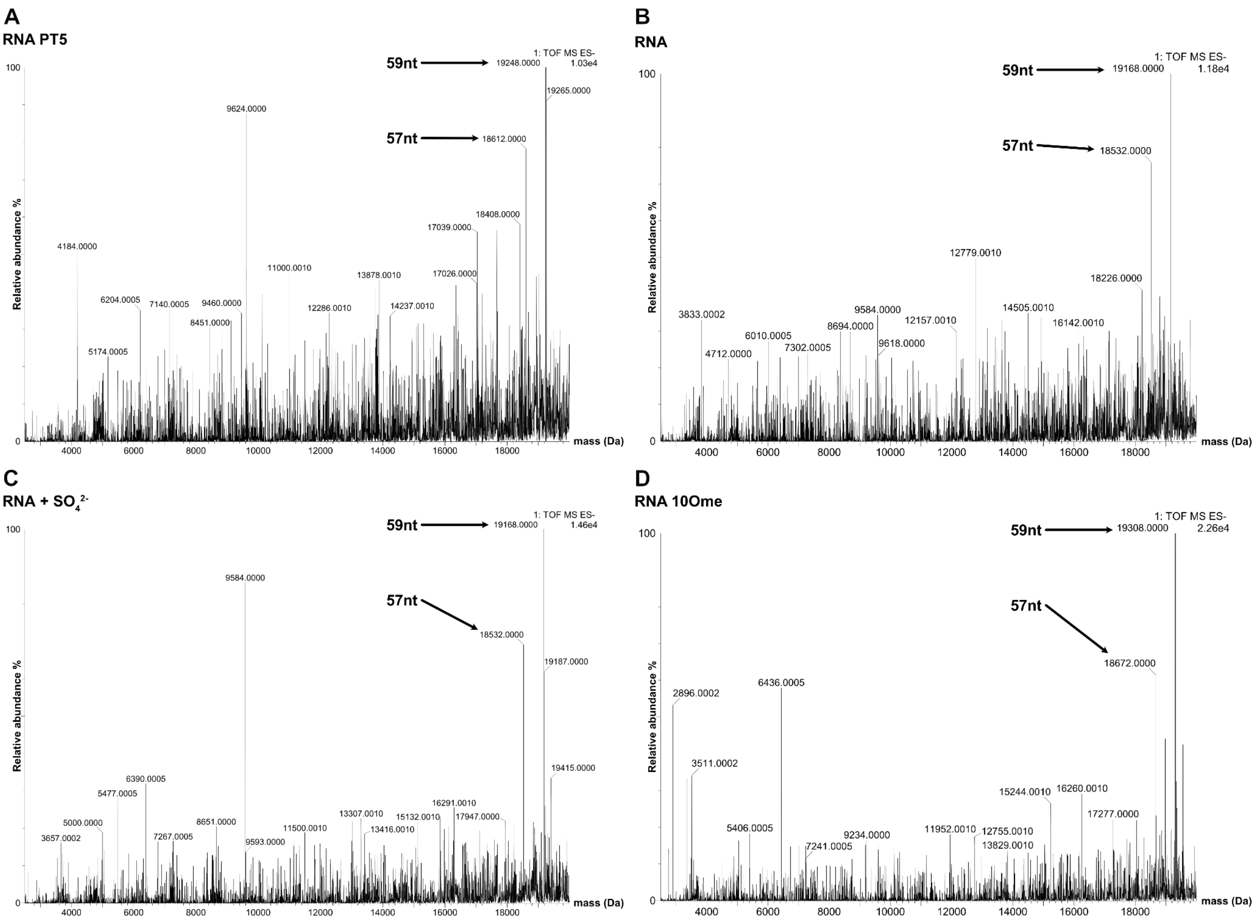

Supplementary Figure 13: LC-MS analysis of 3´-end RNA degradation products after hPNPase digest. Deconvoluted (2,500-20,000 Da) mass spectrum for by hPNPase degraded (**A**) RNA PT5 RNA, (**B**) RNA, (**C**) RNA with 2 mM Na_2SO_4 and 0.1 mM NaH_2PO_4 in the degradation buffer, and (**D**) RNA 10Ome. The first reliably detected degradation product at 57 nt and the full-length RNA with 59 nt are indicated. The theoretical molecular weights for 59n/57nt fragments were calculated to be: PT5 19247.92 Da/18612.54 Da, RNA 19167.92 Da/18532.54 Da, 10Ome 19308.26 Da/18672.89 Da, and RNA + SO_4^{2-} 19167.92 Da/18612.54 Da.

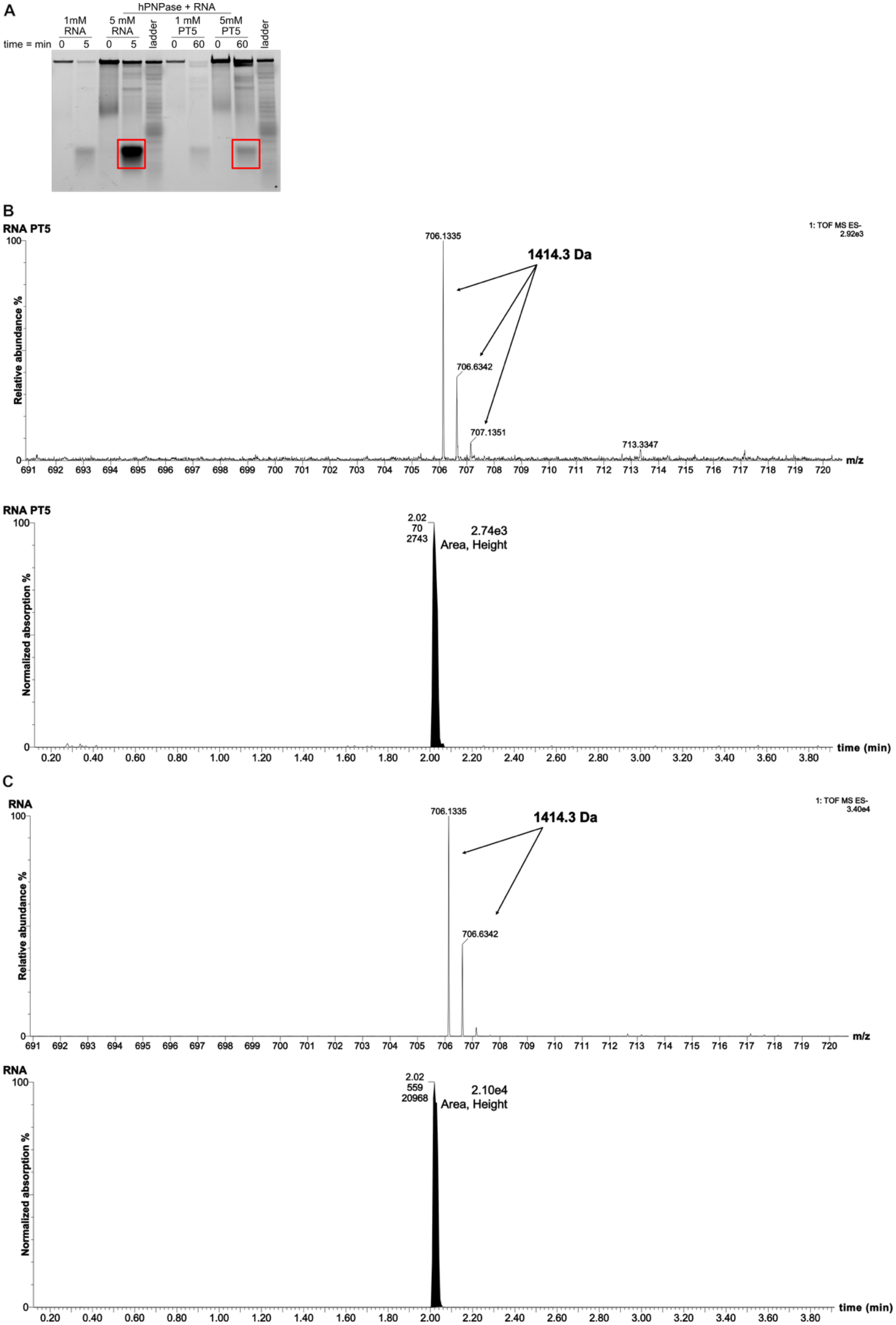
A
hPNPase + RNA
1mM RNA
5 mM RNA
ladder
1 mM PT5
5mM PT5
ladder
time = min 0 5 0 5 0 60 0 60
B
RNA PT5
1: TOF MS ES-
2.92e3
706.1335
1414.3 Da
706.6342
707.1351
713.3347
Relative abundance %
m/z
RNA PT5
2.02
70
2743
2.74e3
Area, Height
Normalized absorption %
time (min)
C
RNA
1: TOF MS ES-
3.40e4
706.1335
1414.3 Da
706.6342
Relative abundance %
m/z
RNA
2.02
559
20968
2.10e4
Area, Height
Normalized absorption %
time (min)

Supplementary Figure 14: LC-MS analysis of 5´-end RNA degradation products after hPNPase digest. (**A**) RNA products after hPNPase digest were analysed using a 15% TBE-Urea PAGE of the LC-MS samples. The smallest detected RNA fragments were further analysed using LC-MS and are highlighted in red rectangles. Analysis of RNA degradation of phosphorothioate RNA (PT5, **B**) and RNA (**C**) using LC-MS. The smallest 5´-RNA fragment detected in the LC-chromatogram and the mass spectrum was 3 nucleotides in size with a theoretical molecular weight of 1415.11 Da, including the 5´-FAM label, and 10 times higher abundance in the RNA compared to the RNA PT5 sample.

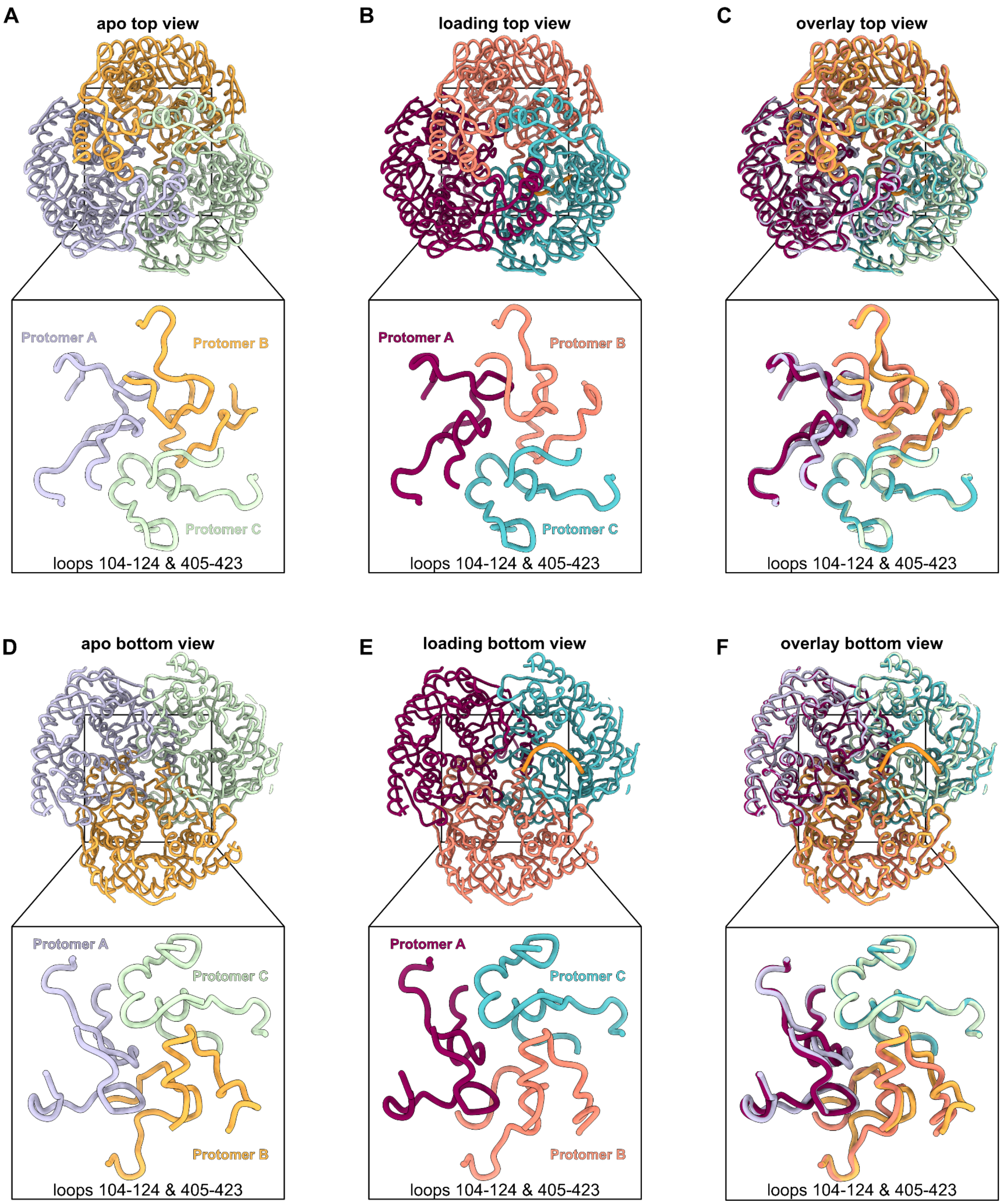

Supplementary Figure 15: Loops comparison between trimeric hPNPase apo loop conformation 1 (hPNPase$^{apo\text{-}loop\text{-}conf1}$; PDB code: 9NJC) and loading (hPNPase^{load}; PDB code: 9NO0). Top view close-up of the pore loops spanning PH1 (residue 104-124) and PH2 (residue 405-423) domains of each protomer in cartoon representation for trimeric hPNPase in (**A**) hPNPase$^{apo\text{-}loop\text{-}conf1}$, (**B**) hPNPase^{load}, or (**C**) an overlay. Each protomer is colored individually in pink, green, and orange for hPNPase$^{apo\text{-}loop\text{-}conf1}$ and violet, blue, and red for hPNPase^{load}. Bottom view close-up of the pore loops spanning PH1 (residue 104-124) and PH2

(residue 405-423) domains of each protomer in cartoon representation for trimeric hPNPase in (**D**) hPNPase$^{apo\text{-}loop\text{-}conf1}$, (**E**) hPNPase^{load}, or (**F**) an overlay. Each protomer is colored individually in pink, green, and orange for hPNPase$^{apo\text{-}loop\text{-}conf1}$ and violet, blue, and red for hPNPase^{load}.

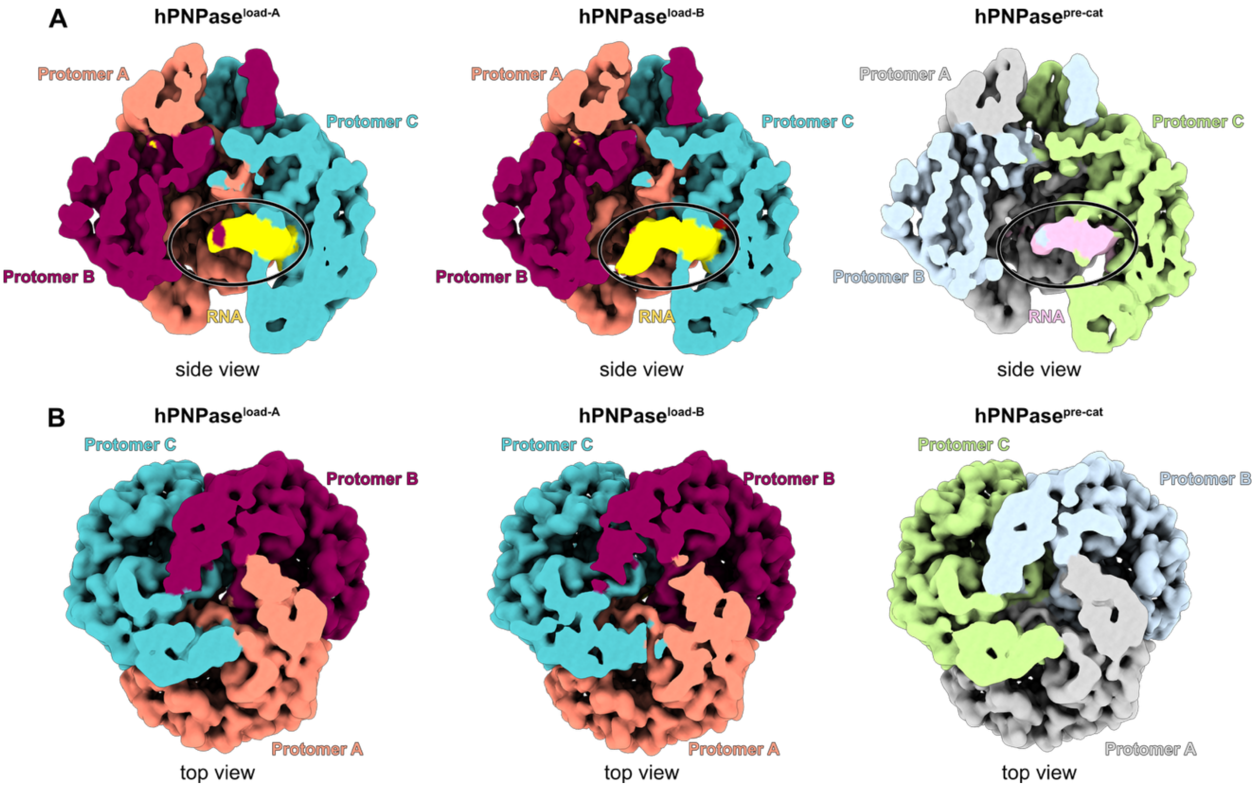

Supplementary Figure 16: Gaussian filtered cryo-EM maps of trimeric hPNPase in RNA loading state A (hPNPase$^{load\text{-}A}$; PDB code: 9NO0) and B (hPNPase$^{load\text{-}B}$; PDB code: 9XZF), and hPNPase in pre-catalytic state (hPNPase$^{pre\text{-}cat}$; PDB code: 9NJB). (**A**) Side, and (**B**) top views of Gaussian-filtered Coulomb potential maps for hPNPase$^{load\text{-}A}$, hPNPase$^{load\text{-}B}$, and hPNPase$^{pre\text{-}cat}$. Each protomer is labelled and colored individually in red, violet, and blue (hPNPase$^{load\text{-}A}$ and hPNPase$^{load\text{-}B}$), or gray, light blue, and green (hPNPase$^{pre\text{-}cat}$). The RNA is highlighted in yellow (hPNPase$^{load\text{-}A}$ and hPNPase$^{load\text{-}B}$) or pink (hPNPase$^{pre\text{-}cat}$).

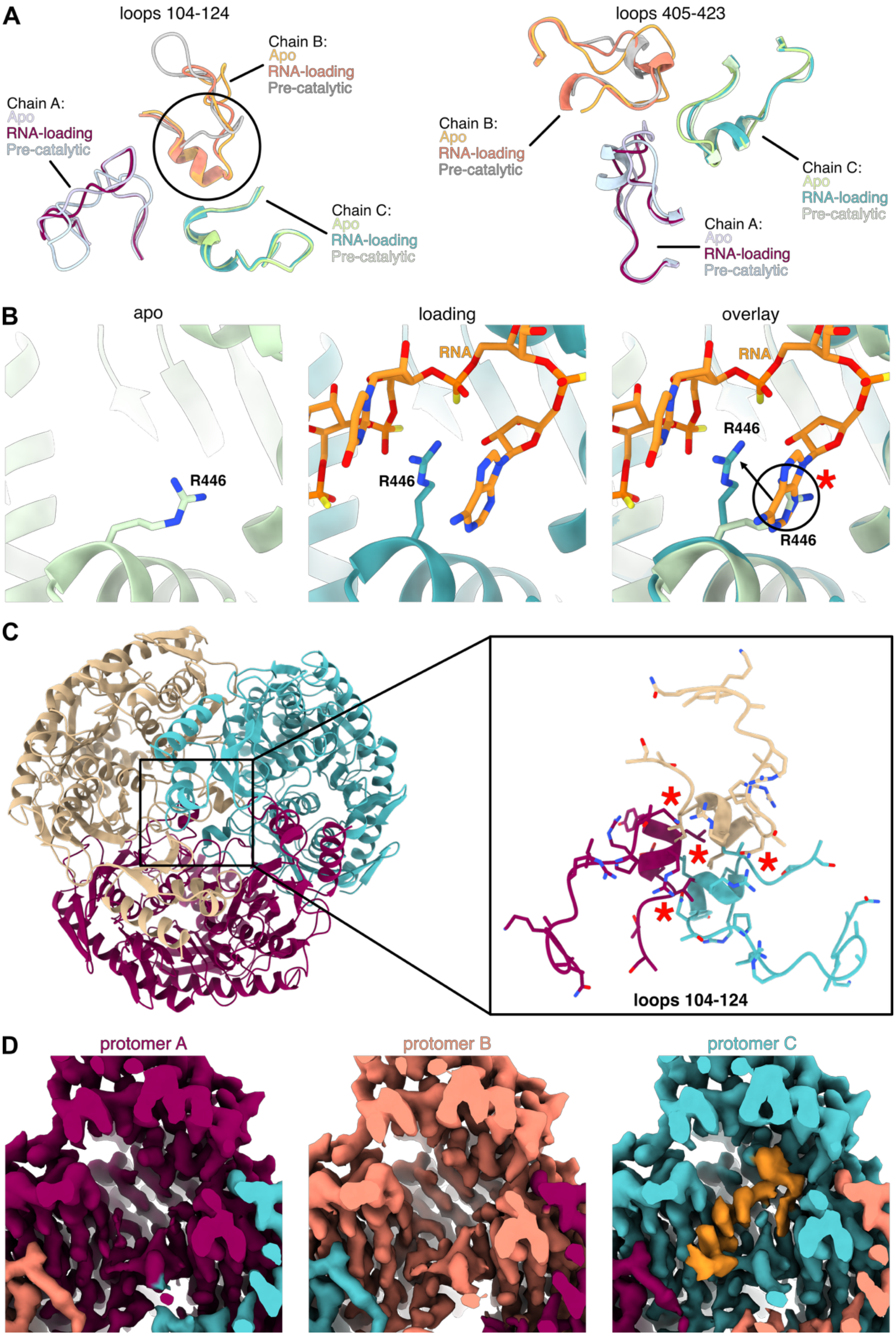
A
loops 104-124
Chain B:
Apo
RNA-loading
Pre-catalytic
Chain A:
Apo
RNA-loading
Pre-catalytic
Chain C:
Apo
RNA-loading
Pre-catalytic
loops 405-423
Chain B:
Apo
RNA-loading
Pre-catalytic
Chain C:
Apo
RNA-loading
Pre-catalytic
Chain A:
Apo
RNA-loading
Pre-catalytic
B
apo
loading
overlay
R446
RNA
R446
RNA
R446
R446
C
loops 104-124
D
protomer A
protomer B
protomer C

Supplementary Figure 17: hPNPase loops and active site change upon RNA binding. (**A**) Comparison of PH1 domain loops 104-124 and PH2 domain loops 405-423 between the hPNPase in apo loop conformation 1 (hPNPase$^{apo\text{-}loop\text{-}conf1}$; PDB code: 9NJC), RNA loading (hPNPase^{load}; PDB code: 9NO0), and pre-catalytic (hPNPase$^{pre\text{-}cat}$; PDB code: 9NJB) state. The structures were superimposed using ChimeraX rigid fitting (35). The most significant structural change between the structures in loops 104-124 in chain B is highlighted. (**B**) The position of residue R446 transitions from a "downward" to an "upward" position from hPNPase^{apo} to hPNPase^{load}. The conformational change is required to accommodate RNA binding and prevent steric clashes. (**C**) The active site-stabilizing protomer B of hPNPase^{load} was aligned with the positions of protomers A and C. Due to steric restraints, only one protomer can adopt the α-helical fold required for active site stabilization. (**D**) Cryo-EM map of trimeric hPNPase^{load} (PDB code: 9NO0). The active sites of each protomer are shown and coloured individually (violet, red, and blue). The bound RNA is highlighted in orange.

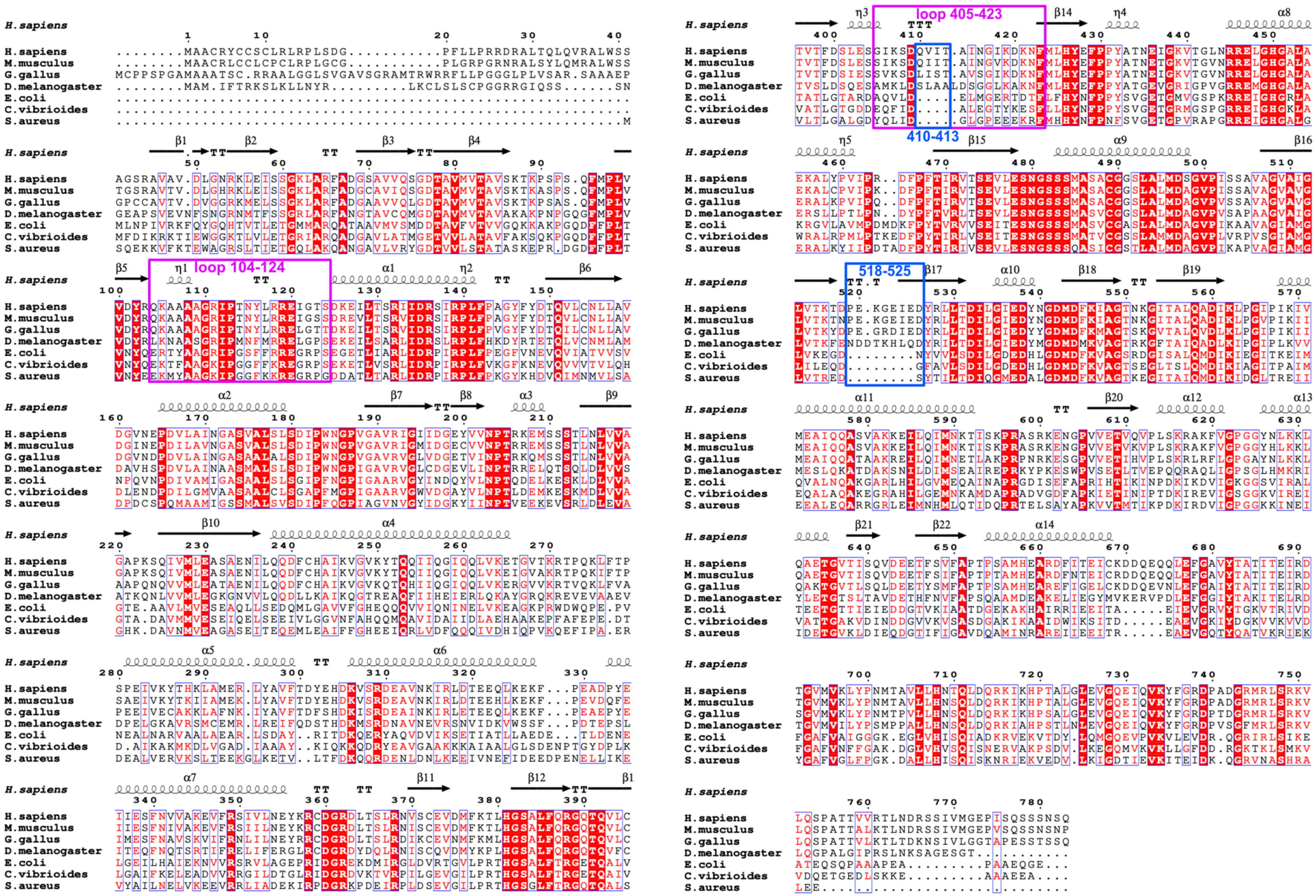
loop 104-124
loop 405-423
410-413
518-525
H.sapiens
M.musculus
G.gallus
D.melanogaster
E.coli
C.vibrioides
S.aureus

Supplementary Figure 18: Sequence alignment between PNPase of different species. The sequences were aligned using PROMALS3D (37) and visualized with ESPript (38). The PH1 and PH2 domain loops are highlighted in blue; the insertions specific for eucaryotic PNPase are highlighted in orange. The UniProt IDs are the following: *H. sapiens* PNPase (Q8TCS8), *M. musculus* (Q3TST0), *G. gallus* (A0A1D5NZ09), *D. melanogaster* (Q5U1D1), *E. coli* (P05055), *C. vinroides* (B8GWZ0), and *S. aureus* (Q2FZ20).

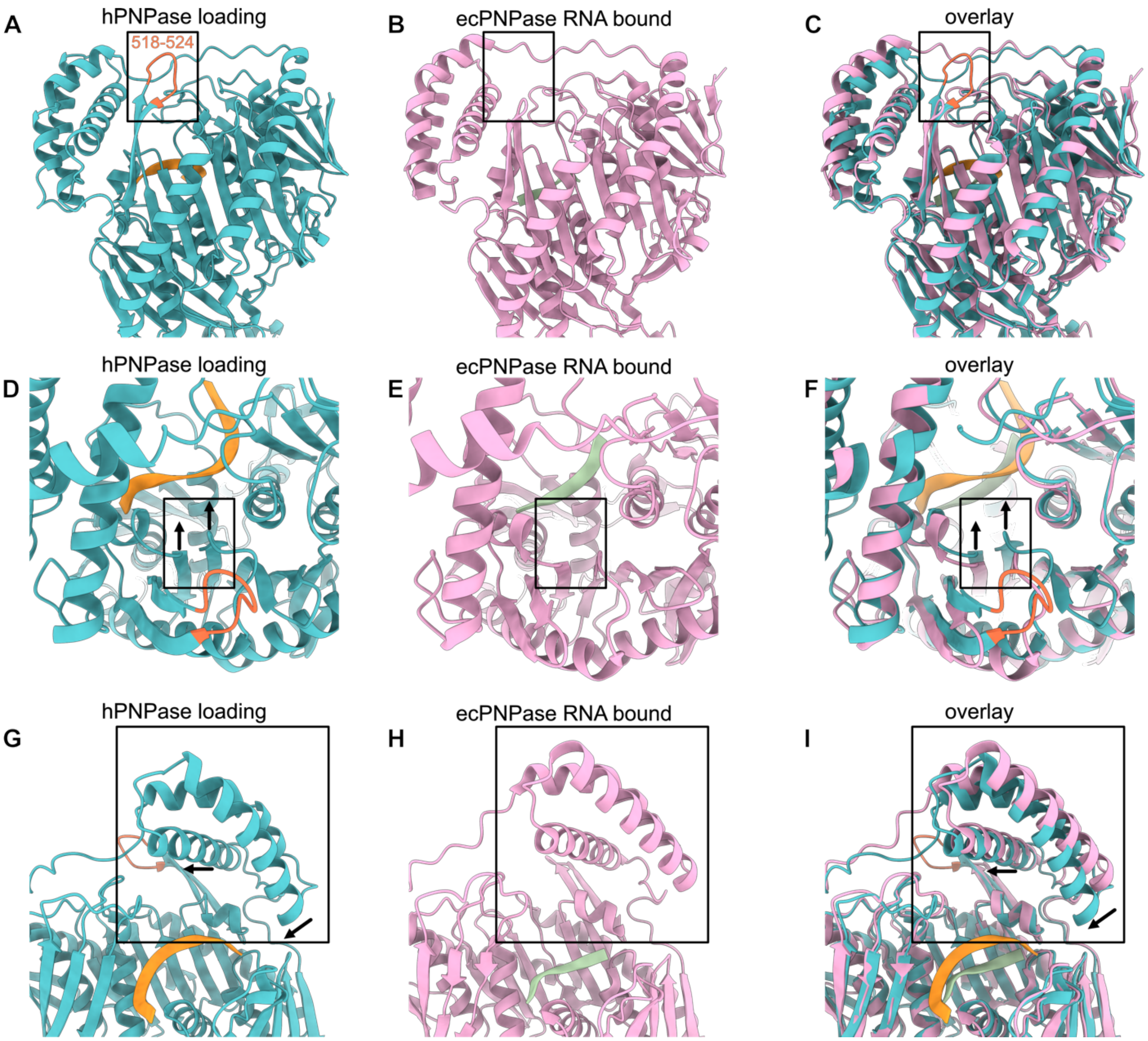

Supplementary Figure 19: Active site structure comparison between human and *E. coli* PNPase. PH2-domain insertion 518-524 is highlighted for human (h) PNPase in RNA-bound loading state (hPNPase^{load}; PDB code: 9NO0) (**A**) and compared to RNA-bound ecPNPase (PDB code: 8VAK) (**B**) individually and as overlay (**C**). Two beta-sheets in the PH2-domain (542-549 and 556-561) are shifted towards the active site in hPNPase^{load} (PDB code: 9NO0) (**D**) compared to RNA-bound (**E**) ecPNPase (PDB code: 8VAK). The structures were overlaid, and for better visualization of the shift in the direction of translation was indicated (**F**). The α-helical domain is oriented and moved towards the active site for hPNPase^{load} (PDB code: 9NO0) (**G**) relative to RNA-bound ecPNPase (PDB code: 8VAK). (**H**) The direction of the translation is indicated as an arrow and visualized in an overlay of the structures (**I**). The structures were superimposed using ChimeraX (35).

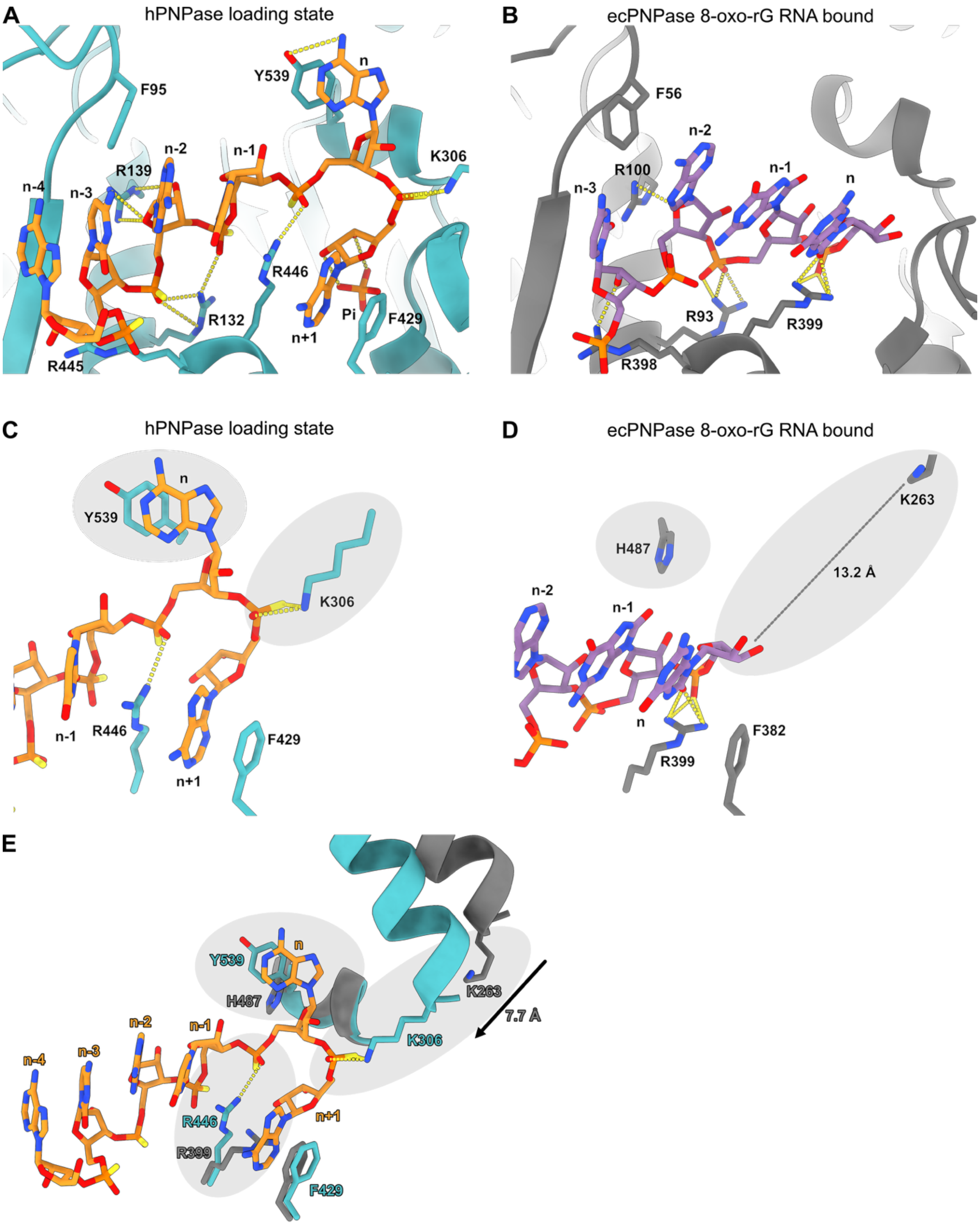

Supplementary Figure 20: Structural comparison of human PNPase in RNA loading state (hPNPase^{load}; PDB code 9NO0) and *E. coli* PNPase bound to 8-oxo-rG RNA (PDB code: 8VAK). (**A**) hPNPase^{load}, key protein-RNA interactions are indicated in dashed lines. (**B**) *E. coli* (ec) PNPase bound to two times 8-oxo-rG modified RNA (PDB code: 8VAK), key protein-RNA interactions are indicated in dashed lines. Structural comparison

between (**C**) hPNPase^{load} and (**D**) ecPNPase bound to double 8-oxo-rG containing RNA (PDB code: 8VAK). Key differences in RNA binding residues are shown for hPNPase^{load} in blue and for ecPNPase in gray. Interactions between hPNPase^{load} or ecPNPase and the orange or violet RNA substrate are indicated in dashed lines. (**E**) Overlay of key differences between hPNPase^{load} and ecPNPase bound to two times 8-oxo-rG modified RNA (PDB code: 8VAK). Residues are shown blue and orange for hPNPase^{load} and gray for ecPNPase. Displacement of the α-helcial domain of hPNPase^{load} relative to ecPNPase is indicated with an arrow. The structures were interposed using ChimeraX (35).

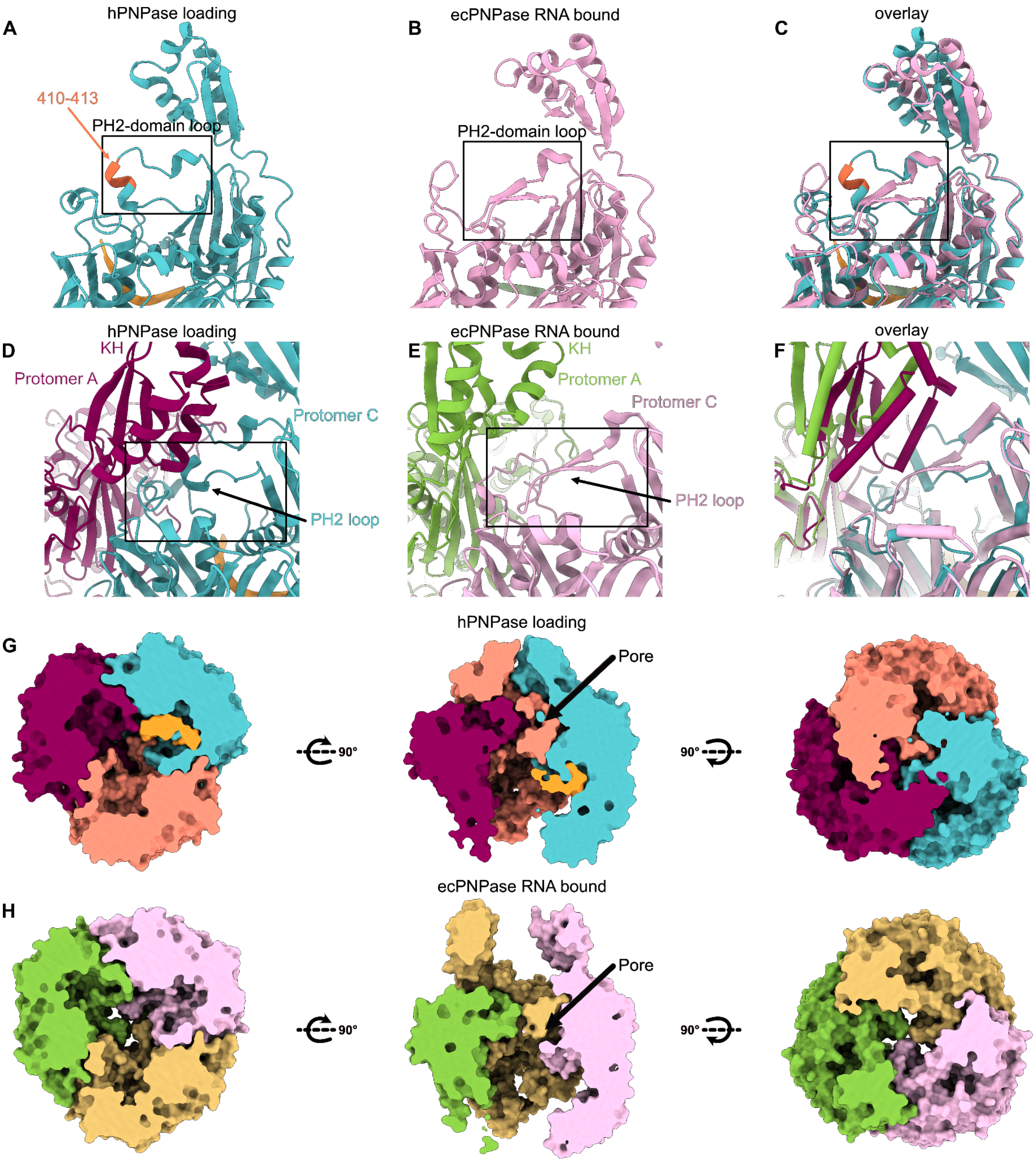

Supplementary Figure 21: Structure alignment between human and *E. coli* PNPase. Comparison of PH2-domain loops between human (h) PNPase in the RNA-bound loading state (hPNPase^{load}; PDB code: 9NO0) (**A**), RNA-bound *E. coli* (ec) PNPase (PDB code: 8VAK) (**B**), and an overlay (**C**). The loop insertion, 410-413, unique to eukaryotic PNPase, is highlighted. The interaction between the PH2-domain loop and KH domain for hPNPase^{load} (**D**), RNA-bound ecPNPase (PDB code: 8VAK) (**E**), and an overlay (**F**). Surface representation highlighting the pore formed by PH1/2 and KH domain as a side, top, and bottom view for hPNPase^{load} (**G**) and RNA-bound ecPNPase (PDB code: 8VAK) (**H**). Each protomer and RNA is colored individually. The structures were interposed using ChimeraX (35).
